# Integrated UHPLC-MS/MS Metabolomics, Network Pharmacology, Molecular Docking, and Experimental Validation Reveal the Anti-Inflammatory Mechanisms of *Pyrus pyrifolia* Peel Extract

**DOI:** 10.3390/antiox15101274

**Published:** 2026-10-02

**Authors:** Ducdat Le, Dong Young Rhyu, Mina Lee

**Affiliations:** 1 College of Pharmacy and Research Institute of Life and Pharmaceutical Sciences, Sunchon National University, Suncheon 57922, Jeonnam-Gwangju, Republic of Korea; ddle@scnu.ac.kr; 2 Department of Natural Cosmetics Science and AI Digital Bioscience Research Institute, Sunchon National University, Suncheon 57922, Jeonnam-Gwangju, Republic of Korea; 3 AI Bio Autonomous Research Core Facility Center, Sunchon National University, Suncheon 57922, Jeonnam-Gwangju, Republic of Korea; 4 School of Food and Pharmaceutical Engineering, Mokpo National University, Mokpo 58554, Jeonnam-Gwangju, Republic of Korea; rhyudy@mokpo.ac.kr; 5 Department of Biomedicine, Health & Life Convergence Sciences, BK21 FOUR, Mokpo National University, Mokpo 58554, Jeonnam-Gwangju, Republic of Korea; 6 Honam Regional Future Drug Development Convergence Education & Research Division (BK21 FOUR), Sunchon National University, Suncheon 57922, Jeonnam-Gwangju, Republic of Korea

**Keywords:** *Pyrus pyrifolia*, UHPLC-MS/MS, network pharmacology, inflammation, TNF, molecular docking

## Abstract

*Pyrus pyrifolia* (Burm. f.) Nakai has long been utilized in traditional East Asian medicine to treat respiratory disorders, fever, cough, and inflammatory conditions. This study aimed to evaluate the antioxidative and anti-inflammatory effects of *P. pyrifolia*. UHPLC-MS/MS analysis revealed that *P. pyrifolia* contained 22 primary and other putative compounds belonging to diverse phytochemical groups—phenolics, flavonoids, fatty acids, terpenoids, alkaloids, and other compounds. Network pharmacology identified a subset of overlapping targets associated with inflammatory diseases, which were significantly enriched in immune- and inflammation-related biological processes. KEGG pathway analysis demonstrated that the Toll-like receptor was the principal pathway potentially regulated by overlapping targets. PPI network analysis revealed TNF to be the most influential hub target, which was verified by molecular docking analysis. Interestingly, the *P. pyrifolia* extract significantly inhibited LPS-induced TNF-α production by modulating IκBα phosphorylation and restoring IκBα expression in the NF-κB signaling pathway. Additionally, the *P. pyrifolia* extract also markedly reduced the phosphorylation of ERK, p38, and JNK, thereby affecting MAPK signaling pathways. Our findings provide mechanistic evidence supporting the traditional medicinal use of *P. pyrifolia* to establish a theoretical framework to develop phytopharmaceuticals for inflammatory diseases.

## 1. Introduction

Inflammation is a fundamental protective response of the immune system that is triggered by pathogens, tissue injury, or harmful stimuli, and its function is to eliminate the causative agent while initiating tissue repair [1]. At the molecular level, pattern recognition receptors, such as Toll-like receptors, detect pathogen- and damage-associated molecular patterns, activating downstream signaling cascades, including the NF-κB and MAPK pathways, that drive the transcription of pro-inflammatory genes [2,3]. Activation of these signaling pathways leads to the synthesis and release of key inflammatory mediators, including cytokines, chemokines, and lipid-derived mediators, such as prostaglandins and leukotrienes [4]. Among the cytokines involved, tumor necrosis factor-alpha (TNF-α), interleukin (IL)-1β, and IL-6 are considered principal drivers of the acute inflammatory response, coordinating leukocyte recruitment, vascular permeability, and the systemic acute-phase response [5]. Prostaglandins, especially PGE_2_, act in concert with these cytokines to amplify local inflammatory signaling and sensitize peripheral nociceptors, contributing to pain and edema at the site of injury [6]. Under normal physiological conditions, acute inflammation is a self-limiting process, resolved through anti-inflammatory mediators such as IL-10 and specialized pro-resolving lipid mediators that restore tissue homeostasis [7]. However, when the initiating stimulus persists or regulatory resolution mechanisms fail, inflammation can transition into a chronic state that is characterized by sustained immune cell infiltration and continuous cytokine production [8]. This chronic inflammatory state is a well-established contributor to the pathogenesis of numerous non-communicable diseases, including atherosclerosis and cardiovascular disease, where oxidized lipoproteins and endothelial activation perpetuate a self-sustaining inflammatory cycle [9]. Similarly, dysregulated cytokine networks involving TNF-α and IL-6 underlie autoimmune and inflammatory disorders such as rheumatoid arthritis [10], while chronic low-grade inflammation has been implicated in metabolic diseases, such as type 2 diabetes, and in tumor initiation and progression [11]. Inflammatory mediators and cytokine signaling play crucial roles in various pathological conditions. As a result, the targeted modulation of key inflammatory factors, such as TNF-α and associated signaling pathways, has emerged as a central strategy in developing anti-inflammatory and disease-modifying therapeutics.

*Pyrus pyrifolia* (Burm. f.) Nakai, known as sand pear, belongs to the *Rosaceae* family. Sand pear is an Asian pear that is widely distributed in Korea and Japan and grows in both temperate and subtropical regions worldwide [12]. Previous studies revealed that this plant is rich in nutrients, sugars, and minerals [13]. Thus, *P. pyrifolia* fruits are consumed fresh or manufactured into products such as juice, jellies, and jams using development methods for natural products [14]. In traditional East Asian medicine, the fruit has been used to treat sputum, cough, hangover, fever, and bowel movement irregularity, while the peel has been used to treat abscesses, cough, dysentery, swelling, skin diseases, and indigestion [15]. Furthermore, previous pharmacological studies have substantiated several of these traditional applications, demonstrating antioxidant, anti-obesity, anti-diabetic, anti-inflammatory, anti-cancer, antibacterial, and hepatoprotective activities [13,16,17,18,19,20]. Chemical investigations have indicated that *P. pyrifolia* has large amounts of bioactive compounds, including phenolics, flavonoids, triterpenoids, arbutin, and alkaloids [19,21,22]. Current pharmacological research on *P. pyrifolia* remains fragmented, lacking a holistic integration of phytochemical profiling, metabolic signaling, and pharmacological evaluation against inflammatory pathologies. To bridge this gap, it is imperative to establish a systematic research framework that synergizes rigorous chemical analysis with the mapping of metabolic pathways. Such a comprehensive approach is essential for elucidating the precise anti-inflammatory mechanisms of *P. pyrifolia*-derived bioactive compounds, thereby providing a robust scientific foundation for its traditional therapeutic applications.

To systematically elucidate the anti-inflammatory potential of *P. pyrifolia*, this study employs an integrated multi-disciplinary approach. We employ high-resolution LC−MS/MS for comprehensive phytochemical profiling, which serves as the foundation for network pharmacology to map the complex interactions between primary metabolites and inflammatory gene targets. To ensure precision, molecular docking is implemented to predict binding affinities and major compounds, providing a strategic orientation for experimental validation. Finally, the therapeutic efficacy is substantiated through TNF-alpha in vitro assays, bridging the gap between computational prediction and biological activity to clarify the molecular mechanisms of *P. pyrifolia* peel. Additionally, antioxidative assays were further conducted to investigate the antioxidative effects of *P. pyrifolia* peel.

## 2. Materials and Methods

### 2.1. Extraction and Preparation of Sample

*P. pyrifolia* peel was collected in Naju, Jeonnam, Republic of Korea, on 15 October 2023. A voucher specimen (SCNUP50) was deposited in the Laboratory of Pharmacognosy, College of Pharmacy, Sunchon National University (Suncheon, Republic of Korea). *P. pyrifolia* peel was dried at 35 °C for 48 h and then extracted by using distilled water for 4 h at 60 °C. The extract was filtered through Whatman No. 2 filter paper (Cytiva, Hangzhou, China) to remove the particles. The extract solution was evaporated under a vacuum rotary evaporator at 40 °C before being completely dried by a vacuum concentrator (Savant SpeedVac SPD2030, Thermo Fisher Scientific, Waltham, MA, USA). This dried *P. pyrifolia* extract was stored in the refrigerator for further analysis. An accurate amount of dried extract was accurately weighted and dissolved in 50% methanol, followed by filtering through a polytetrafluoroethylene membrane (size of 0.22 μm, manufactured by Agilent Technologies, Santa Clara, CA, USA). Then, an LC-MS/MS system was employed to analyze the sample, and data acquisition and compound annotation were conducted following our previous method [23].

### 2.2. Determination of Compound and Inflammation-Related Targets

The selected compounds were input into Pubchem (https://pubchem.ncbi.nlm.nih.gov; accessed on 12 May 2026) to collect their SMILES. Then, we imported the SMILES into the Swisstargetprediction web platform (https://www.swisstargetprediction.ch/predict.php; accessed on 26 May 2026) to retrieve the targets. On the other hand, the inflammation-related targets were retrieved from the GeneCards web platform at https://www.genecards.org (accessed on 10 June 2026) by inputting the key word “inflammation”, followed by setting filter factors, including human as the species and a score above 5. All compound and disease targets were standardized through the UniProt database (https://www.uniprot.org/, accessed on 16 June 2026) [24]. A Venn diagram was used to identify intersecting targets between compound targets and disease targets.

### 2.3. Gene Topology Enrichment Using Metascape and SHINYGO 0.85 Databases

Enrichment analysis of the overlapping targets was further analyzed using Metascape (version v3.5.20260701) and SHINYGO 0.85 databases using the human species setting. Functional enrichment analysis was carried out on both Metascape and ShinyGO to systematically explore the biological significance of these targets. The analysis included Gene Ontology (GO) enrichment analysis, including biological processes (BP), molecular functions (MF), and cellular components (CC), as well as Kyoto Encyclopedia of Genes and Genomes (KEGG) pathway enrichment analysis. The aim was to find significantly enriched signaling pathways and functional terms. Enrichment analysis revealed statistical robustness, with the integration of multiple authoritative biological databases enabled by the Metascape platform. Functional annotations were performed by ShinyGO, and the results of the enrichment analysis were intuitively validated and visualized. Then, significantly enriched GO terms and KEGG pathways were identified at a threshold of *p* < 0.01 after multiple-testing correction.

### 2.4. Construction of Protein–Protein and Plant-Compound–Target–Disease Interaction Networks

To construct the protein–protein interaction (PPI) network, 146 overlapping targets were imported into the STRING database (version 12.0; https://string-db.org; accessed on 11 June 2026) with the organism restricted to Homo sapiens and a minimum required interaction confidence score of 0.7. Disconnected nodes were removed, and the resulting interaction data were exported and imported into Cytoscape (version 3.10.4) for visualization and topological analysis. Network parameters, including degree, betweenness centrality, and closeness centrality, were calculated using the Cytoscape Cytohubba plugin to identify hub proteins that may play central roles in the underlying biological processes. In parallel, a compound–target–disease network was constructed to visualize the relationships among the selected compounds of *P. pyrifolia*, their protein targets, and inflammation-associated genes, illustrating the potential multi-target, multi-pathway mechanism of action of *P. pyrifolia* against inflammation.

### 2.5. Molecular Docking In Silico

The protein structure (PDB ID: 2AZ5) was obtained from the protein data bank (https://www.rcsb.org/; accessed on 2 July 2026). The 3D structures of selected compounds (3, 4, 6–8, 10–14, 26, 30–32, 35–37, 77, 85, 117–119, dexamethasone) were collected from the Pubchem database. The MGL tool (version 1.5.7) was used to prepare the receptor and ligands. Prior to docking the selected compounds, the native ligand was separated from the protein structure, which was prepared by removing crystallized water molecules and heteroatoms. Then, the protein was added with hydrogen atoms at its polar part and Kollman charges. The ligand was prepared by adding Gasteiger charges. Grid box dimensions were set by centering on the original native ligand-binding site to cover the entire catalytic pocket. Binding energies were calculated using the autodock4.2 algorithm. This docking procedure was implemented prior to docking the native ligand (307: 6,7-dimetho 6,7-dimethyl-3-[(methyl{2-[methyl({1-[3-(trifluoromethyl)phenyl]-1H-indol-3-yl}methyl)amino]ethyl}amino)methyl]-4H-chromen-4-one) to the receptor. The result showed that the RMSD value of crystallographic conformations between the original and redocked native ligand was 0.027 Å (<2.0 Å), indicating that the docking protocol is reliable. Pymol and Discovery studio 2021 software were used to visualize the interactions of the ligand and residues at the binding pocket of the protein [25].

### 2.6. Biological Assays

#### 2.6.1. DPPH and ABTS Assays

The antioxidative properties of the *P. pyrifolia* extract were assessed through DPPH and ABTS radical scavenging activities using our previous method [26] without modification. The sample and positive control (AA: ascorbic acid) were prepared at concentrations of 10 and 100 μg/mL.

#### 2.6.2. Cell Culture

The macrophage cell line RAW 264.7 was purchased from the Korean Cell Line Bank (Seoul, Republic of Korea). Cells were cultured in Dulbecco’s modified Eagle’s medium (DMEM) supplemented with 10% heat-inactivated fetal bovine serum (FBS) and 100 IU/mL penicillin (HyClone, Logan, UT, USA) solutions. The cultures were maintained at 37 °C in a humidified incubator under a 5% CO_2_ atmosphere [27].

#### 2.6.3. Cell Viability

After seeding the cells (2 × 10^5^) into 96-well plates in the culture medium and incubating them for 24 h, the cells were pretreated with samples for 2 h at concentrations of 10 and 100 μg/mL before stimulation with LPS (100 ng/mL) for 20 h. Cell survival was evaluated using the MTT (3-[4,5-dimethyl-2-thiazolyl]-2,5-diphenyl tetrazolium bromide) (Sigma-Aldrich, Saint Louis, MO, USA) assay. The cultured cells were incubated with MTT at 37 °C for 4 h. The supernatant was then aspirated, and 100 µL of DMSO was added to each well. The absorbance of the formazan crystals was checked at 570 nm with a microreader (BioTek Instruments, Winooski, VT, USA) [27].

#### 2.6.4. Measurement of TNF-α Production

To determine the effect of samples on TNF-α production in LPS-stimulated RAW264.7 cells, we conducted an experiment following our previous method. Briefly, cells were seeded at 2 × 10^5^ cells/well in a 96-well plate. After treatment with samples (either dexamethasone or extract at concentrations of 10 and 100 µg/mL), cells were incubated for 2 h. They were then stimulated with LPS and incubated further. After 24 h, the TNF-α production in LPS-activated RAW264.7 cells was measured by using the ELISA kit (BD OptEIATM, San Jose, CA, USA), following the manufacturer’s instructions [27].

#### 2.6.5. Evaluation of Effects of *P. pyrifolia* Extract on NF-κB and MAPK Signaling Pathways

Mouse macrophages were seeded at a density of 2 × 10^5^ cells/well [27] in 6-well plates in DMEM (2 mL) and incubated for 24 h. Seeded cells were pretreated with samples for 2 h and stimulated with LPS (100 ng/mL). The whole-cell lysates were extracted with a protein extraction solution (ProPrep, iNtRON, Biotechnology, Daejeon, Republic of Korea) for 30 min. The protein concentration was determined by the Bradford method (Pierce™ Bradford Protein Assay Kit, Waltham, MA, USA). Proteins (20 µg) were separated by SDS-PAGE using 10% acrylamide gels and transferred to a PVDF membrane. Antibodies, including P-p44/42 MAPK (#9101), p44/42 (#4695), P-p38 (#9211), p38 (#9212), JNK (#4668), P-JNK (#9252), and p-IκBα (#9246), were procured from Cell Signaling Technology (Danvers, MA, USA). The IκBα (SC-1643) antibodies were obtained from Santa Cruz Biotechnology Inc. (Santa Cruz, CA, USA). β-Actin (AB_2289199) was purchased from BD Bioscience (San Jose, CA, USA). After being transferred to PVDF membranes, they were blocked for 2 h with 5% skim milk before incubation with the primary antibodies diluted 1:1000 in 2.5% skim milk and incubated at 4 °C for 18 h, and the blots were washed three times with Tween20/Tris-buffered saline (T/TBS) and incubated with an HRP-conjugated secondary antibody diluted 1:2000 in 5% skim milk for 2 h at room temperature, followed by three additional washes with T/TBS. The expression of the targeted protein was detected using Super Signal™ West Femto Maximum Sensitivity Substrate (Thermo Fisher Scientific, Waltham, MA, USA). Protein bands were captured using a bio-imaging system (MicroChemi 4.2 Chemiluminescence System, DNR Bio-Imaging Systems, Neve Yamin, Israel).

### 2.7. Statistical Analysis

Data were expressed as mean ± standard deviation (S.D.) of three replicates. The observed differences were statistically analyzed using one-way ANOVA and Duncan’s multiple range tests in GraphPad Prism software version 8.0 (GraphPad Software, Inc., San Diego, CA, USA). Results with * *p* < 0.05 and ** *p* < 0.01 were declared statistically significant.

## 3. Results

### 3.1. Chemical Profiling of P. pyrifolia by UHPLC-MS/MS

**Figure 1:**
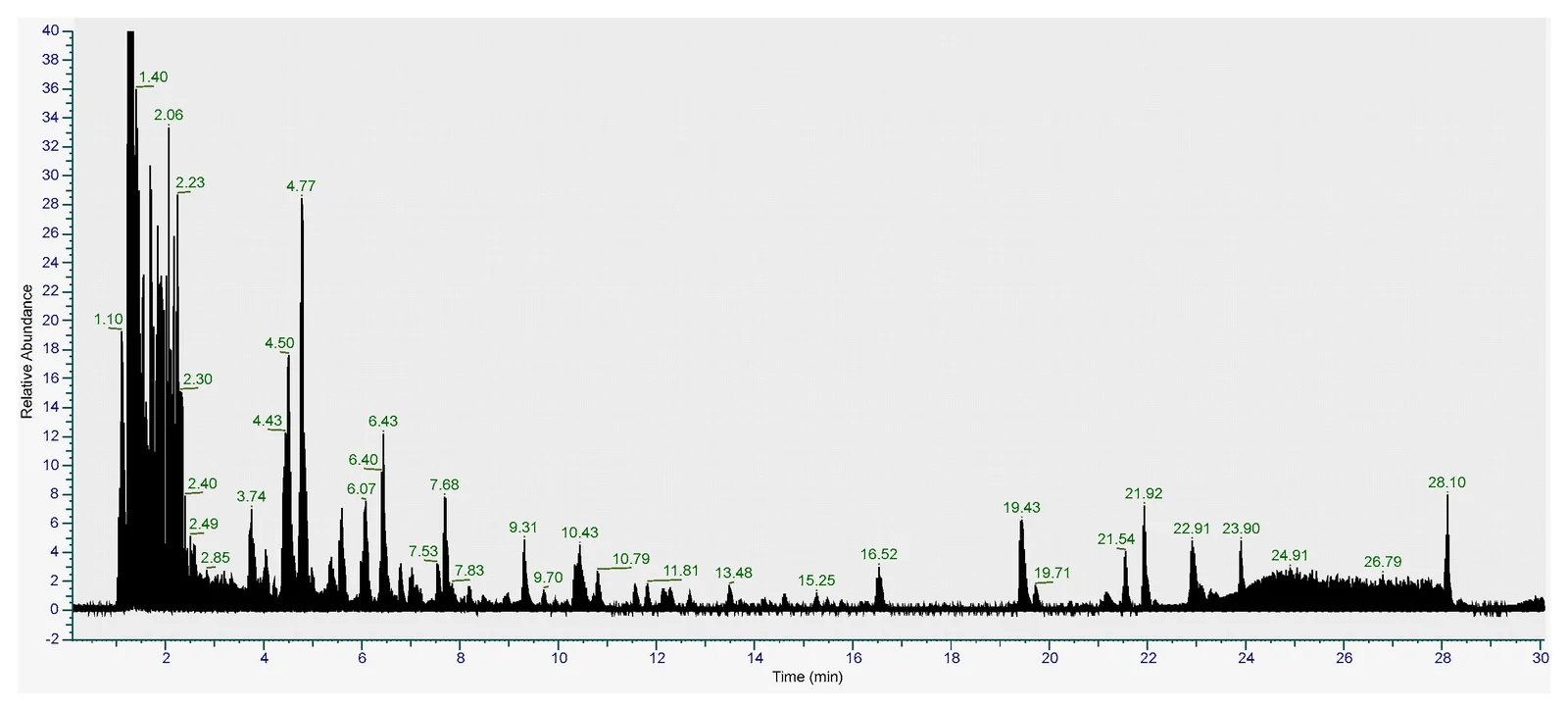
Chemical profile of *P. pyrifolia* extract obtained using a UHPLC-MS/MS system.

Comprehensive phytochemical profiling of the peel extract of *P. pyrifolia* was performed using a platform coupling ultra-high-performance liquid chromatography with tandem mass spectrometry (UHPLC-MS/MS) to characterize its chemical constituents. Figure 1 illustrates that the chromatographic separation generated a complex metabolite profile consisting of numerous well-resolved peaks distributed across the chromatographic gradient, indicating the presence of structurally diverse secondary metabolites. The acquired MS/MS spectra enabled the annotation of 120 chemical constituents (Table S1, Supporting Information) through the analysis and comparison of experimental mass precursors and isotopes with fragmentation patterns and available spectral databases. Among them, 22 major compounds were identified as primary constituents of the peel extract by integrating their peak areas detected by chemical chromatography (Table S2, Supporting Information).

### 3.2. Identification of Putative Anti-Inflammatory Targets

To systematically investigate the molecular mechanisms underlying the anti-inflammatory activity of *P. pyrifolia*, a network pharmacology strategy was employed by integrating phytochemical-derived targets with inflammation-associated disease targets. The workflow enabled the identification of overlapping genes that potentially mediate the therapeutic effects of *P. pyrifolia* against inflammatory diseases.

**Figure 2:**
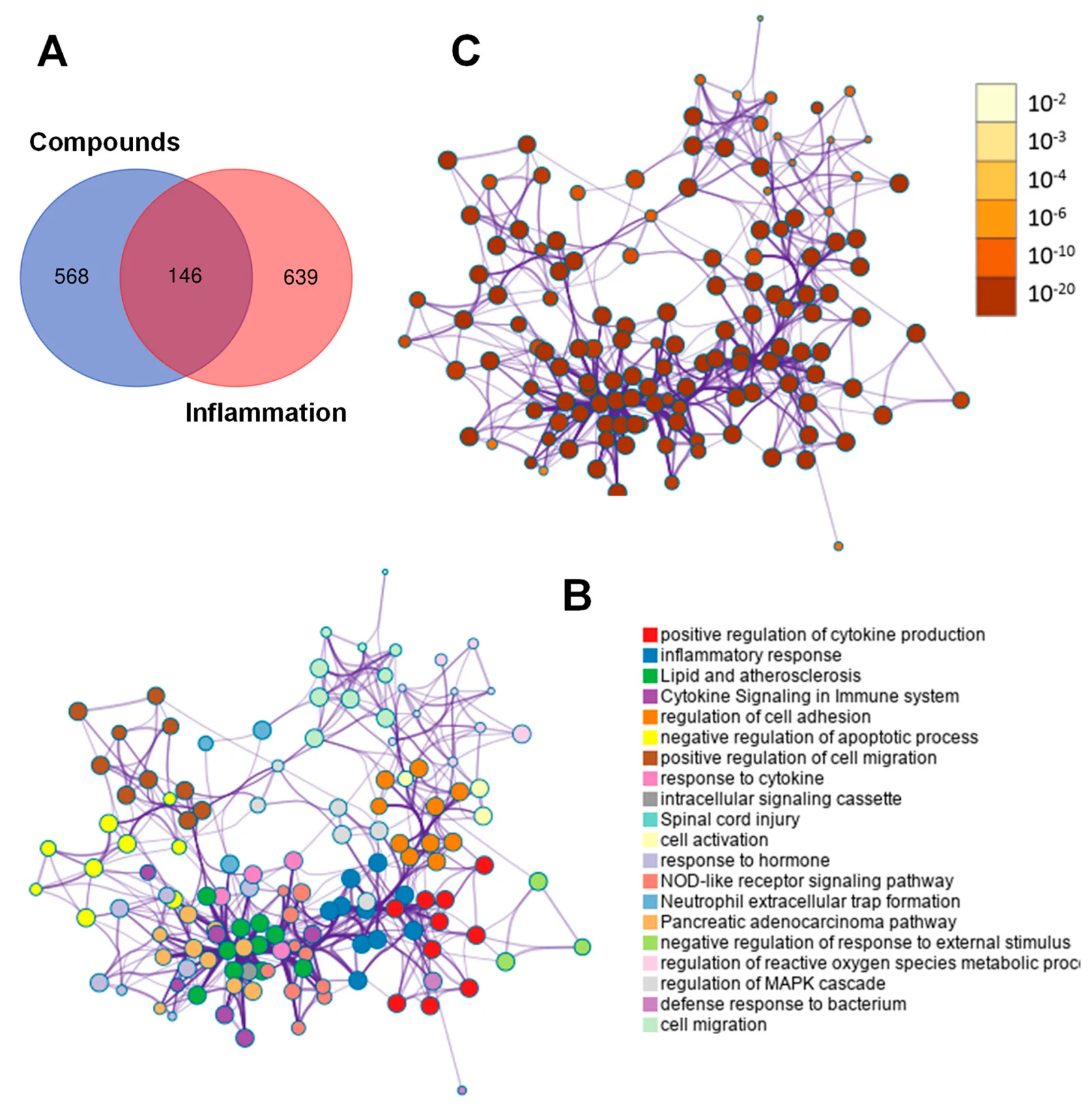
Gene Ontology and pathway enrichment analyses of the overlapping targets. (**A**) The Venn diagram illustrates the intersecting targets between compound and inflammation disease-related targets. (**B**) Enriched tocology clusters of overlapping targets. (**C**) Statistical analysis of Gene Ontology clusters based on *p* values (the darker the color, the more statistically significant the node).

Figure 2A shows that target predictions for the identified phytochemicals generated an extensive list of candidate proteins, while inflammation-related targets were collected from DisGENET databases. Comparative analysis using a Venn diagram revealed a subset of 146 intersecting genes (Figure 2A) shared between compound and inflammation-related targets, indicating that these proteins may represent the principal molecular mediators through which *P. pyrifolia* exerts its pharmacological effects.

The overlapping targets were subsequently subjected to functional enrichment analysis to determine their biological significance using Metascape web tools. Gene Ontology (GO) clustering demonstrated that these common targets were predominantly enriched in biological processes associated with inflammatory regulation, immune responses, cytokine-mediated signaling, cellular responses to external stimuli, and regulation of intracellular signal transduction (Figure 2B). These enriched biological processes collectively suggest that the identified targets participate in the complex molecular network governing inflammatory progression. Visualization of the enriched GO clusters further highlighted the interconnected nature of the biological processes associated with the overlapping targets (Figure 2C). Highly significant clusters exhibited greater statistical confidence and formed tightly connected functional modules, implying that rather than being independently activated, multiple inflammatory signaling events are regulated in coordination. The identification of these intersecting targets provides important evidence supporting the multi-component and multi-target characteristics of *P. pyrifolia*. The overlap between phytochemical targets and inflammation-associated genes therefore suggests that the bioactive constituents of *P. pyrifolia* may collectively interfere with several critical regulatory nodes involved in inflammatory pathogenesis.

Moreover, the functional characteristics of the overlapping targets indicate their involvement in immune cell activation, cytokine production, inflammatory mediator synthesis, and intracellular communication among immune signaling pathways. These findings provide an important mechanistic basis for understanding the ethnopharmacological application of *P. pyrifolia* in managing inflammatory conditions and for justifying subsequent analyses of signaling pathway enrichment and protein interaction networks.

### 3.3. Gene Ontology and KEGG Pathway Enrichment Analyses

**Figure 3:**
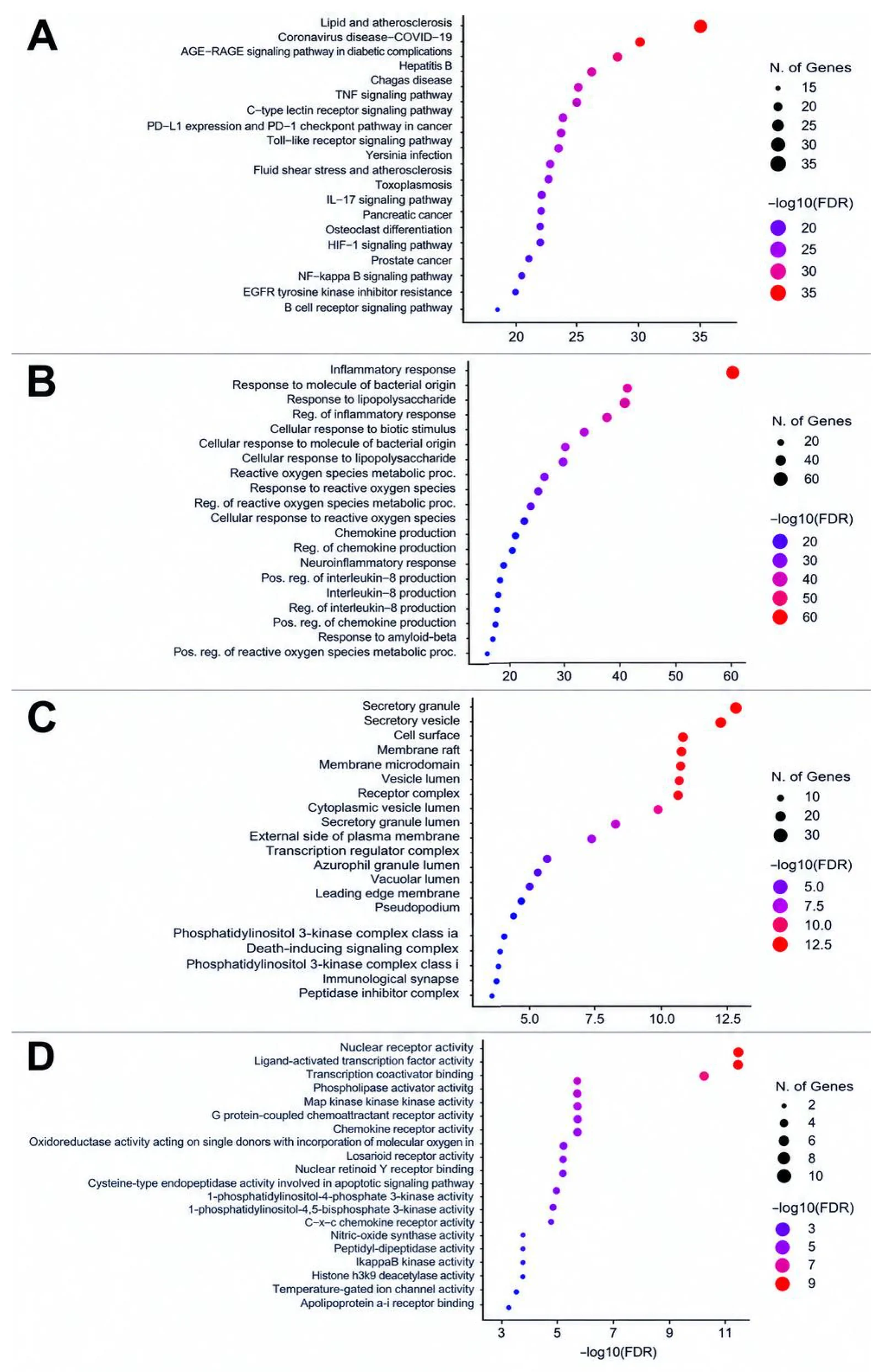
Top 20 ranked enrichment terms of KEGG pathways (**A**) and Gene Ontology [biological process (**B**), cellular component (**C**), and molecular function (**D**)].

To further elucidate the biological functions and molecular pathways associated with the overlapping targets, GO and KEGG pathway enrichment analyses were conducted. These analyses provide comprehensive insights into biological processes, cellular localization, molecular functions, and signaling pathways potentially regulated by the bioactive compounds identified in *P. pyrifolia*. The KEGG enrichment analysis identified numerous inflammation-related pathways among the significantly enriched signaling networks (Figure 3A). Several of these pathways are recognized as central regulators of innate and adaptive immune responses, suggesting that the therapeutic activity of *P. pyrifolia* may involve the coordinated modulation of multiple inflammatory cascades. The enrichment profile demonstrated that the overlapping targets participate in pathways that are responsible for cytokine signaling, immune receptor activation, cellular stress responses, and inflammatory mediator production. These pathways collectively constitute the molecular framework governing inflammatory initiation and progression.

Gene Ontology enrichment analysis further classified the overlapping targets into three functional categories (BP, CC, and MF). Within the biological process category, the top enriched terms primarily involved the regulation of inflammatory response, cytokine production, immune system processes, cellular responses to stress, regulation of signal transduction, and positive regulation of transcription associated with inflammatory signaling (Figure 3B). These biological functions indicate that the identified targets play essential roles in coordinating immune activation and maintaining inflammatory homeostasis. Analysis of the cellular component category revealed that most of the overlapping proteins were localized to cellular compartments closely associated with signal transduction and immune regulation, including plasma membrane, membrane rafts, cytosol, receptor complexes, and intracellular signaling complexes (Figure 3C). Such localization is consistent with their participation in receptor-mediated signaling events initiated by inflammatory stimuli.

The molecular function analysis demonstrated significant enrichment of protein binding, kinase activity regulation, cytokine receptor binding, transcription factor binding, enzyme regulator activity, and signaling receptor interactions (Figure 3D). These molecular functions further support the hypothesis that the phytochemicals identified in *P. pyrifolia* may interfere with multiple molecular interactions involved in inflammatory signal amplification.

### 3.4. Identification of Key Inflammatory Signaling Pathways

To further elucidate the molecular mechanisms underlying the anti-inflammatory activity of *P. pyrifolia*, the overlapping targets were mapped to significantly enriched KEGG pathways. Among the identified pathways, the Toll-like receptor (TLR) and tumor necrosis factor (TNF) signaling pathways (Figure S1 of Supplementary Information) emerged as two of the most prominently enriched inflammatory cascades, indicating that these pathways may serve as the principal pharmacological targets of the bioactive constituents identified in the extract.

### 3.5. Protein–Protein Interaction Network Analysis Identifies TNF as a Central Hub Target

**Figure 4:**
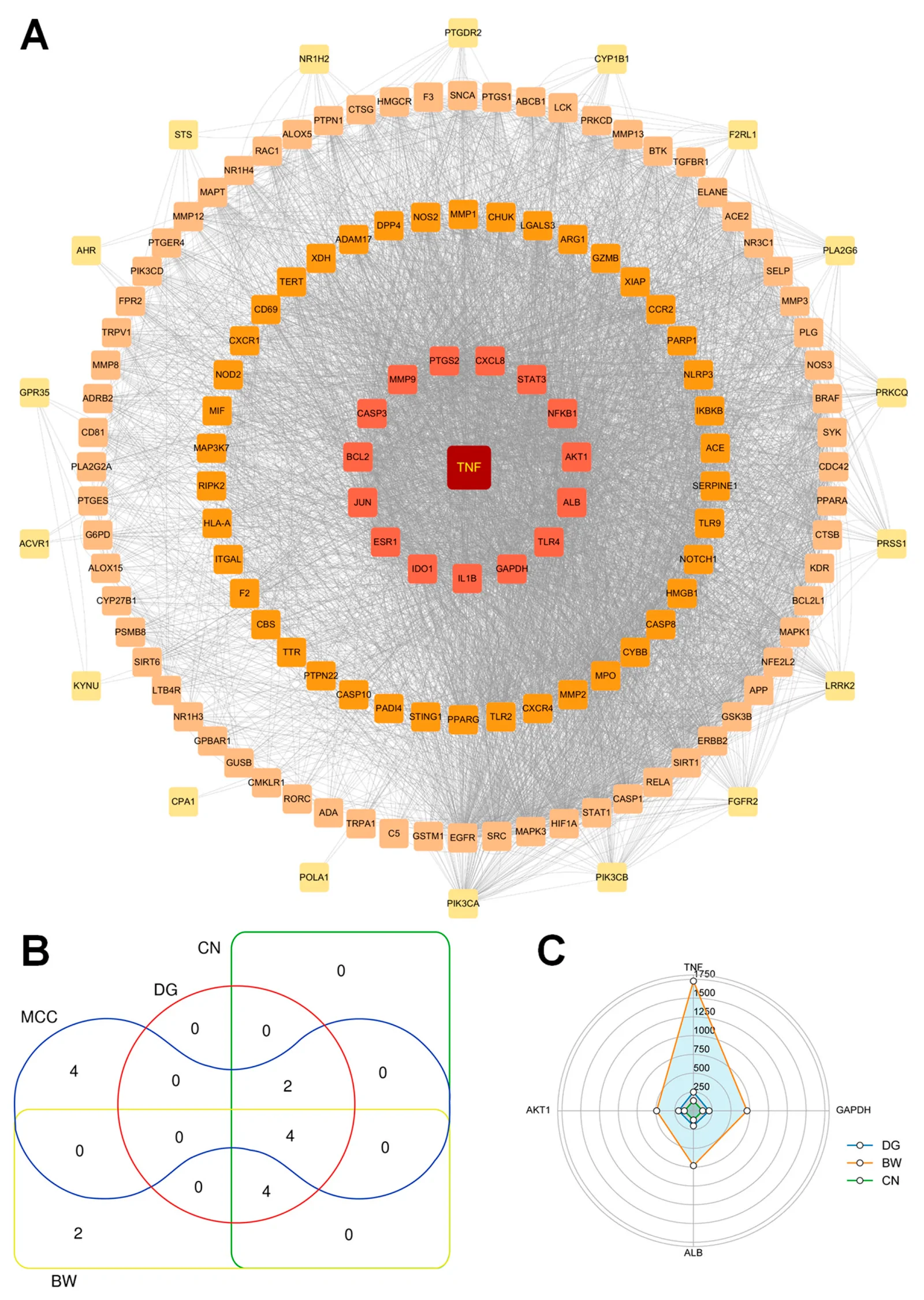
Interaction network of overlapping targets. (**A**) Protein–protein interaction network of intersecting targets. (**B**) Identification of hub targets based on network topology analysis of degree (DG), closeness (CN), betweenness (BW), and maximal clique centrality (MCC) scores. (**C**) TNF targets showing the highest scores.

To identify the most influential proteins involved in the anti-inflammatory mechanism of *P. pyrifolia*, the overlapping targets were subjected to PPI network analysis. After removing two unconnected nodes, construction of the interaction network revealed an extensively interconnected protein network comprising numerous inflammation-associated proteins, suggesting that the therapeutic effects of the phytochemicals are mediated through the coordinated regulation of multiple molecular interactions (Figure 4A). The PPI network included 144 nodes along with 5550 edges, with an average of 38.52 neighbors per node, and exhibited a dense interaction architecture in which several proteins occupied highly connected positions, indicating their essential roles in maintaining network integrity and coordinating inflammatory signaling. To quantitatively identify the most important nodes within the network, topological analyses were performed with the top ten nodes using four complementary centrality algorithms (DG, CC, BC, and MCC). These algorithms evaluate different aspects of network organization, including the number of direct interactions, communication efficiency, information flow, and overall network connectivity. As presented in Figure 4B, six inflammation-related proteins were consistently identified based on all four topological parameters, demonstrating their critical regulatory roles within the interaction network. Among all candidate targets, TNF achieved the highest overall topological score and occupied the most central position within the interaction network (Figure 4C). This finding indicates that TNF functions as the principal regulatory hub connecting the numerous inflammatory signaling pathways identified in the present study. Given its extensive interactions with cytokines, transcription factors, immune receptors, and intracellular kinases, TNF thereby likely represents one of the primary molecular mediators through which *P. pyrifolia* exerts its pharmacological effects.

### 3.6. Biological Function Analysis Reveals TNF-Centered Regulation of Inflammatory Responses

**Figure 5:**
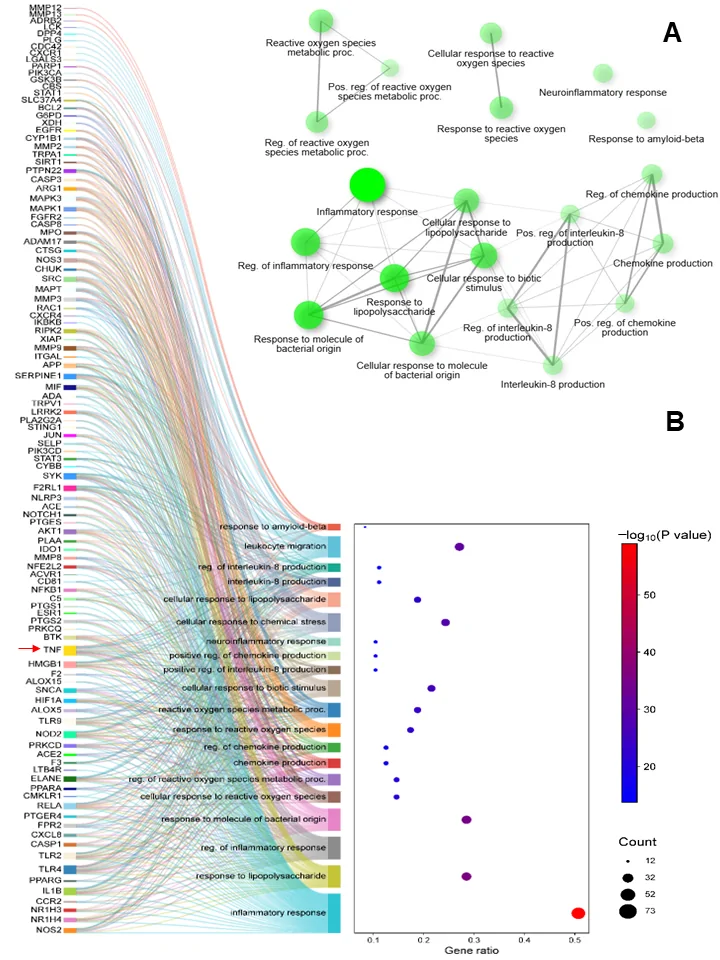
Biological functions and interaction network of overlapping targets associated with inflammatory diseases. (**A**) Network visualization showing significantly enriched biological processes linked to inflammatory disease pathogenesis. (**B**) Sankey plot suggesting a high number of interactions of the TNF gene (red arrow) with inflammatory disease.

To further characterize the biological significance of overlapping targets, we constructed a biological function enrichment network, which provides a comprehensive overview of the biological processes regulated by overlapping targets and illustrates how these processes cooperate to mediate inflammatory responses. Figure 5A shows that the enriched biological processes formed highly interconnected functional modules, suggesting that the anti-inflammatory activity of *P. pyrifolia* is mediated through the coordinated regulation of multiple biological events molecular mechanisms. To further investigate the relationships between key genes and inflammatory diseases, a Sankey plot was constructed to visualize the associations among overlapping targets and biological functions (Figure 5B). The Sankey analysis revealed that TNF exhibited the greatest degree of connectivity with inflammation-related biological processes and disease nodes, indicating that this gene occupies a central position within the inflammatory network. TNF was linked to numerous downstream biological functions involved in immune activation, cytokine production, inflammatory signaling, and cellular communication, emphasizing its pivotal role in orchestrating inflammatory responses.

The prominent position of TNF within the Sankey network strongly corroborates the findings obtained from the protein–protein interaction analysis, in which, based on multiple topological algorithms, TNF was identified as the highest-ranking hub protein. Moreover, the inflammatory pathways also suggest that modulation of TNF-α cytokine production may influence numerous downstream signaling pathways, thereby producing widespread anti-inflammatory effects. The consistency between independent analytical approaches considerably strengthens the reliability of TNF as the principal molecular target underlying the anti-inflammatory activity of *P. pyrifolia*.

Therefore, TNF was chosen to investigate its potential interaction with representative compounds using a molecular docking approach.

### 3.7. Construction of the Compound–Target–Disease Interaction Network

**Figure 6:**
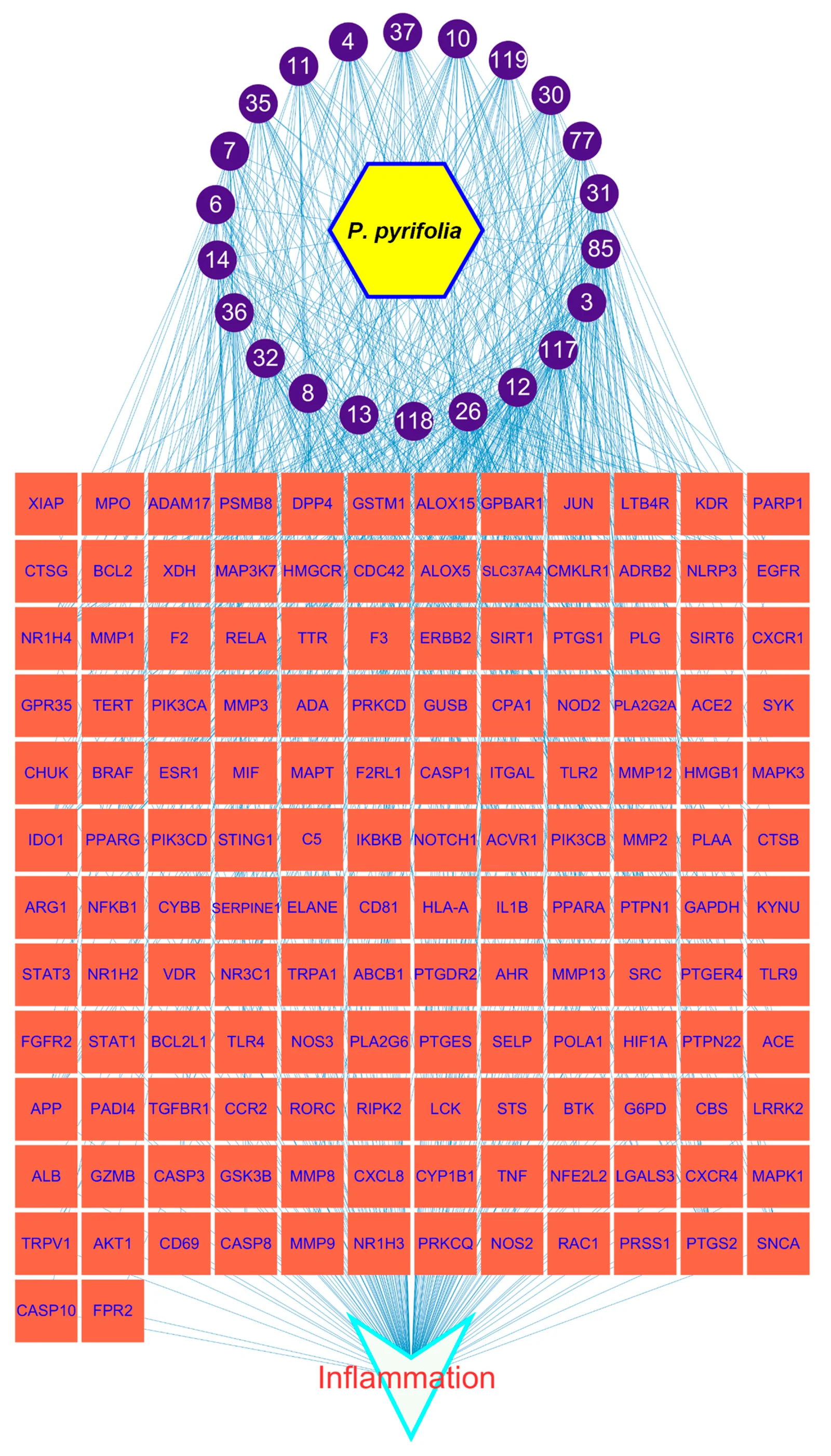
Plant-compound–target–disease interaction network showing the compounds identified in *P. pyrifolia* interacting with targets related to inflammatory diseases.

To comprehensively visualize the complex pharmacological relationships among phytochemicals, molecular targets, and inflammatory diseases, a plant-compound–target–disease interaction network was constructed. Figure 6 illustrates the network, which integrates the identified phytochemicals from *P. pyrifolia* with their predicted protein targets and inflammation-associated disease nodes, providing a systems-level overview of the potential therapeutic mechanisms of the extract. The resulting network exhibited the typical “component–target–disease” architecture that is widely recognized as a defining characteristic of traditional herbal medicines. Individual phytochemicals were connected to multiple protein targets, while each target simultaneously interacted with numerous compounds, indicating substantial pharmacological redundancy and functional complementarity among the constituents of *P. pyrifolia*. Such interaction patterns suggest that the therapeutic activity of the extract is unlikely to depend on a single bioactive compound but instead arises from the collective actions of chemically diverse metabolites.

Furthermore, numerous target proteins were linked to multiple inflammatory disease nodes, indicating that these proteins represent common molecular regulators underlying different inflammatory disorders. This observation highlights the possibility that *P. pyrifolia* possesses broad-spectrum anti-inflammatory potential through the modulation of shared pathogenic mechanisms, rather than disease-specific targets.

Network topology also revealed considerable variation in node connectivity among the identified compounds. Certain phytochemicals exhibited extensive interactions with numerous target proteins, suggesting that these compounds may contribute more substantially to the overall pharmacological activity of the extract. Conversely, several targets served as convergence points for multiple phytochemicals, reinforcing the concept of synergistic regulation, in which structurally distinct metabolites cooperatively influence the same signaling proteins.

Importantly, the interaction network demonstrated that the major hub proteins identified by the PPI analysis were simultaneously targeted by multiple constituents of *P. pyrifolia*. This convergence provides additional support for the hypothesis that network-level pharmacological regulation, rather than single-target inhibition, underlies the anti-inflammatory effects of the extract. Such cooperative interactions may enhance therapeutic efficacy while minimizing the likelihood of resistance or compensatory activation of alternative inflammatory pathways.

Collectively, the compound–target–disease network provides a comprehensive systems pharmacology framework linking the chemical composition of *P. pyrifolia* to its predicted biological functions.

By integrating phytochemical identification with target prediction and disease association, the network illustrates how structurally diverse natural products may collectively regulate interconnected inflammatory signaling pathways. These findings further strengthen the mechanistic evidence supporting the ethnopharmacological use of *P. pyrifolia* and provide a theoretical basis for future experimental studies aimed at validating the biological activities of its major bioactive constituents.

### 3.8. Molecular Docking Validates the Interactions Between Representative Phytochemicals and the Hub Target

**Figure 7:**
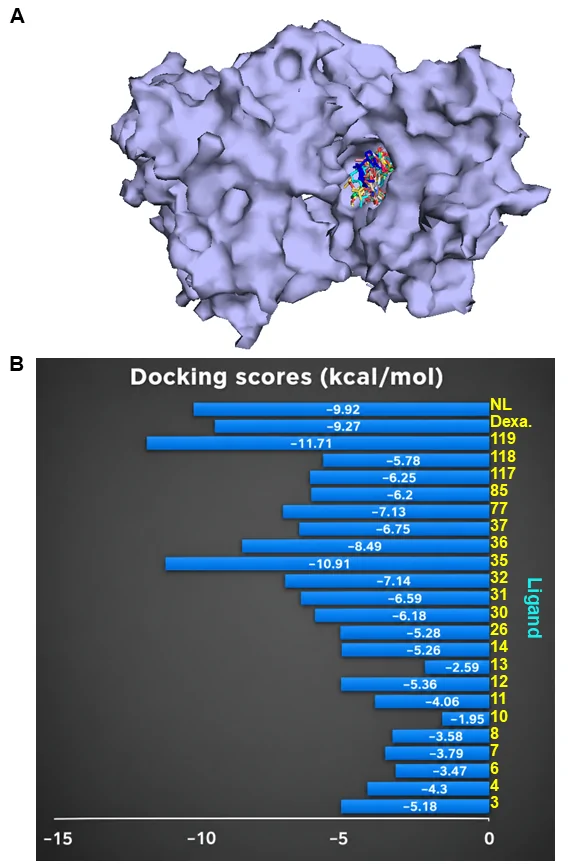
Visualization of molecular docking of compounds to the target protein. (**A**) Selected compounds and dexamethasone docked into the same region of the native ligand of the protein. (**B**) Binding affinities of compounds.

To further verify the interactions predicted by network pharmacology analysis, molecular docking simulations were performed to evaluate the binding affinities of representative compounds with target protein. Molecular docking provides structural evidence supporting ligand–protein interactions and their binding affinities. As illustrated in Figure 7, primary compounds identified in *P. pyrifolia*, along with the positive control (dexamethasone), were docked into the active site of the TNF protein (Figure 7A), surrounded by interactions with active residues of protein.

Visualization of the docking complexes revealed that the compounds established multiple covalent interactions with residues at the active pocket, including hydrogen bonds, hydrophobic interactions, van der Waals contacts, and π-related interactions. Analysis of binding affinity further demonstrated that the investigated compounds possessed favorable docking scores (Figure 7B). Among them, compounds 35 and 119 exhibited docking energies of −10.91 and −11.71 kcal/mol, respectively, with stronger binding than dexamethasone or the native ligand (−9.27 or −9.92 kcal/mol, respectively), suggesting that these compounds are naturally occurring constituents of *P. pyrifolia* that possess considerable potential to modulate inflammatory effects. Indeed, compounds 35 and 119 formed hydrogen bonds with key residues, including SER60, LEU120, GLY121, and TYR151, of the protein (17). In addition, both compounds displayed some hydrophobic interactions with amino acids in the binding pose of the protein. Compounds 3, 12, 14, 26, 30–32, 36, 37, 77, 85, 117, and 118 showed moderate binding, with docking scores ranging from −5.18 to −8.49 kcal/mol. These compounds showed hydrogen bonds with SER60, GLN61, LEU120, GLY121, and TYR151 amino acids and were surrounded by key residues at the binding pocket of the protein (Figure S1, Supplementary Information). The docking results indicate that the *P. pyrifolia* extract may exert an anti-inflammatory effect through coordinated interactions with TNF-associated signaling pathways.

### 3.9. Inhibitory Effect of P. pyrifolia Extract

**Figure 8:**
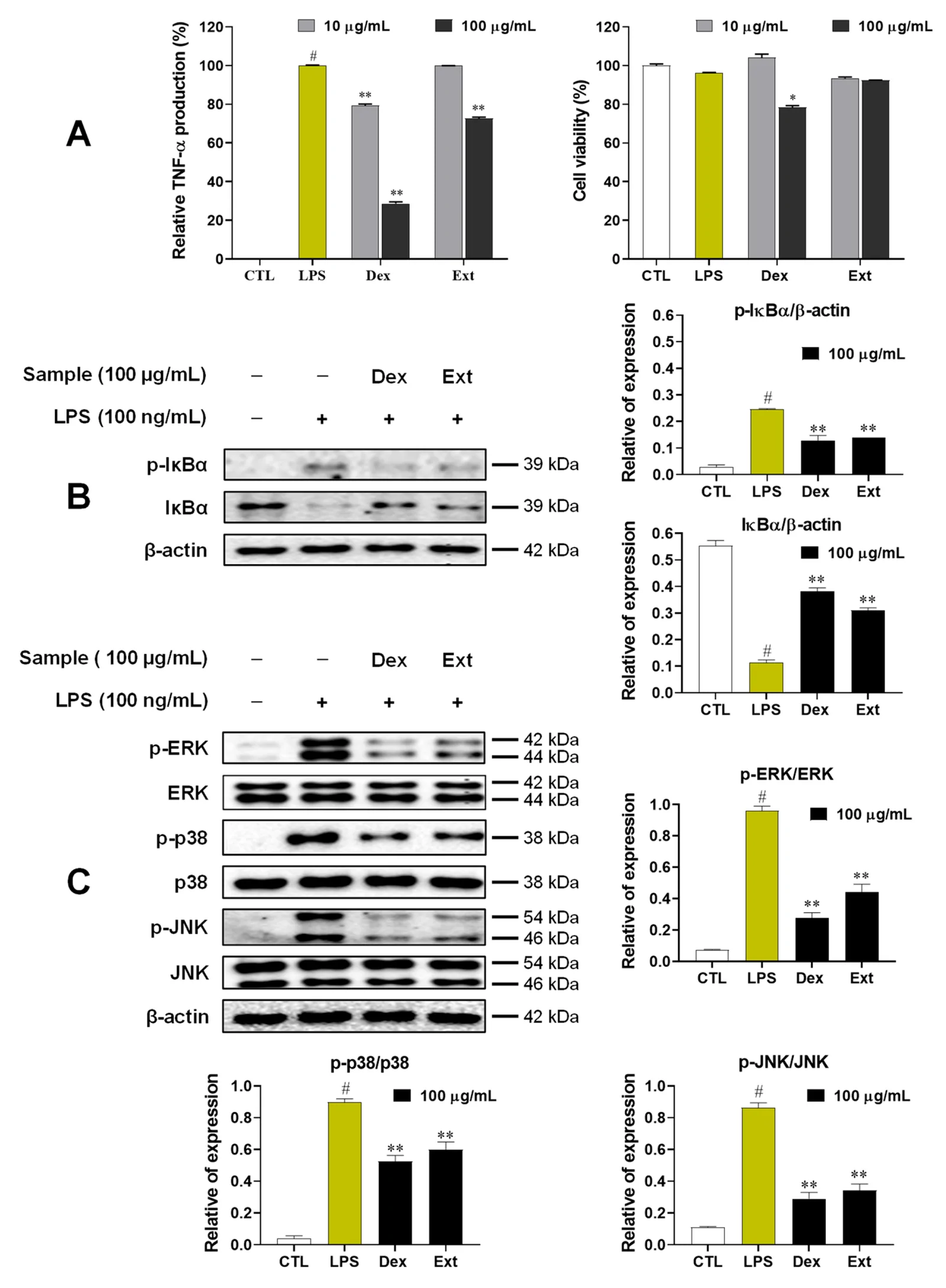
Molecular mechanisms of *P. pyrifolia* extract toward NF-κB and MAPK signaling pathways. (**A**) Inhibitory and cytotoxic effects of extract. (**B**) Representative Western blots of phospho-IκBα (p-IκBα, 39 kDa), total IκBα (39 kDa), and β-actin (42 kDa, internal control), along with relative quantification of p-IκBα and IκBα. (**C**) Representative Western blots of phospho-ERK (p-ERK, 42/44 kDa), total ERK (42/44 kDa), phospho-p38 (p-p38, 38 kDa), total p38 (38 kDa), phospho-JNK (p-JNK, 54/46 kDa), total JNK (54/46 kDa), and β-actin (42 kDa, internal control), along with relative quantification of p-ERK/ERK, p-p38/p38, and p-JNK/JNK. Protein expression levels were normalized to β-actin and/or their respective total protein and are expressed relative to the control group (*n* = 3). Data are presented as mean ± SD. # *p* < 0.05 vs. CTL group; * *p* < 0.05, ** *p* < 0.01 vs. LPS treatment without sample addition.

Based on this computational prioritization, we conducted experiments to assess the anti-inflammatory effect of the *P. pyrifolia* extract by evaluating its ability to inhibit TNF-α production induced by LPS-stimulated RAW264.7 cells. Figure 8A shows that the level of TNF-α production was significantly different between the control (not treated with LPS) and LPS-treated cells. This observation indicated that LPS successfully activated TNF-α production in RAW264.7 cells. After cells were treated with the *P. pyrifolia* extract, TNF-α production was reduced compared to that in LPS-treated cells without sample treatment at both concentrations of 10 and 100 μg/mL. Therefore, the *P. pyrifolia* extract suppressed TNF-α production in LPS-stimulated RAW264.7 cells.

### 3.10. P. pyrifolia Extract Suppresses IκBα NF-κB Signaling Pathway

To elucidate the molecular mechanism generated by the network pharmacology analysis, the *P. pyrifolia* extract was examined for its effect on the IκBα NF-κB signaling pathway in LPS-stimulated RAW264.7 cells. Figure 8B demonstrates that LPS stimulation successfully activated the NF-κB cascades by increasing the phosphorylation of IκBα (p-IκBα) and decreasing the expression of the IκBα protein compared to the untreated control. Pretreatment with the *P. pyrifolia* extract significantly suppressed LPS-induced IκBα phosphorylation and restored total IκBα expression in a manner comparable to that in the positive control (Dex: Dexamethasone). These results suggest that the extract exerts its anti-inflammatory effect by inhibiting IκBα phosphorylation and subsequent degradation, thereby preventing the activation and nuclear translocation of NF-κB.

### 3.11. P. pyrifolia Extract Suppresses MAPK Signaling Pathways

To investigate whether the anti-inflammatory activity of the *P. pyrifolia* extract involves modulation of the MAPK signaling pathway, the phosphorylation levels of ERK, p38, and JNK were examined by Western blotting in LPS-stimulated cells. Stimulation with LPS markedly increased the phosphorylation of ERK, p38, and JNK compared with the untreated control. These observations confirmed activation of all three MAPK cascades. Treatment with the *P. pyrifolia* extract significantly attenuated LPS-induced phosphorylation of ERK (p-ERK/ERK), p38 (p-p38/p38), and JNK (p-JNK/JNK), with inhibitory effects comparable to those of the positive control (dexamethasone). Thus, these findings indicate that the *P. pyrifolia* extract exerts its anti-inflammatory effect by suppressing the activation of all three major MAPK signaling pathways (ERK, p38, and JNK), which likely contributes, together with its inhibitory action on IκBα/NF-κB signaling, to the overall attenuation of LPS-induced inflammatory responses (Figure 8C).

### 3.12. Antioxidative Effect of P. pyrifolia Extract

Network pharmacology analysis suggested that *P. pyrifolia* exerted an antioxidative effect among biological functions, although it was not very pronounced. Therefore, we also evaluated the antioxidative effect of the *P. pyrifolia* extract based on its radical scavenging activity. The results indicated that *P. pyrifolia* displayed some radical scavenging activity against DPPH and ABTS free radicals. However, the antioxidative effect was not significant compared to that of the positive control (ascorbic acid) (Figure S1, Supplementary Information).

## 4. Discussion

*P. pyrifolia*, which is traditionally valued in folk medicine, has demonstrated extensive anti-inflammatory potential [13] across various experimental models, validating its historical use to treat respiratory and gastrointestinal ailments. Previously, a *P. pyrifolia* extract demonstrated anti-inflammatory effects that were mediated through the inhibition of pro-inflammatory cytokines, mediators, enzymes, and oxidative stress pathways. In gastrointestinal studies, fruit extracts and cellulose nanofibers have been shown to alleviate DSS-induced colitis by suppressing the activation of inflammatory cells like leukocytes, decreasing interleukin-1beta (IL-1β) levels, and reducing myeloperoxidase (MPO) activity [28]. Furthermore, the *P. pyrifolia* extracts possess significant respiratory benefits, reducing airway sensitivity and suppressing IgE and Th2-type cytokines (IL-4, IL-5, and IL-13) in asthma models [29]. Beyond systemic effects, the water extracts of this plant have exhibited protective properties against UVA-induced skin inflammation and allergic responses by downregulating the mRNA expression of IL–1β, IL–6, IL–2, and TNF-α [30]. Importantly, these anti-inflammatory agents are often significantly more concentrated in the fruit peel than in the flesh, suggesting that the whole fruit or targeted peel extracts provide the greatest therapeutic efficacy [31].

Metabolomic analysis in this study revealed annotations of 120 compounds in the water extract of *P. pyrifolia* peel. Among them, 20 primary constituents were further investigated for their potential in treating inflammation. To elucidate the therapeutic mechanisms underlying the anti-inflammatory potential of the compounds under study, network pharmacology analysis was performed to identify overlapping targets and their associated biological functions. The Venn diagram in Figure 2A shows that of the 714 compound-related targets and the 785 inflammation-related targets, 146 targets intersected, representing the putative candidates through which these compounds may exert their anti-inflammatory effects. Gene Ontology and pathway enrichment analyses of these 146 overlapping targets through the Metascape database revealed a diverse and biologically coherent network of processes that are closely linked to inflammatory disease pathogenesis (Figure 2B). The enriched clusters were predominantly associated with immune and inflammatory regulation, including positive regulation of cytokine production, inflammatory response, cytokine signaling in the immune system, and response to cytokines [32]. Notably, clusters related to lipid and atherosclerosis and MAPK cascade regulation further underscore the interconnection between metabolic dysregulation and chronic inflammatory processes [33,34]. The prominence of these clusters suggests that the overlapping targets converge on core mechanisms of immune cell activation, cytokine-mediated signal transduction, and leukocyte trafficking, all of which are hallmark features of inflammatory disease progression. Statistical validation of the enrichment results (Figure 2C) demonstrated that most enriched terms and pathways were highly significant, with node color intensity (*p*_values > 0.01) indicating that the densely interconnected, darker-colored nodes correspond to the most statistically robust functional clusters. The high degree of connectivity observed among these significant nodes further supports the notion that the compounds act through a multi-target, multi-pathway mechanism rather than a single dominant pathway, consistent with the network pharmacology paradigm.

To further characterize the biological relevance of the 146 overlapping targets, KEGG pathway and GO enrichment analyses were performed, encompassing BP, CC, and MF categories. KEGG pathway enrichment (Figure 3A) revealed that the overlapping targets were most significantly enriched in canonical inflammatory signaling cascades, such as the TNF signaling pathway, Toll-like receptor signaling pathway, IL-17 signaling pathway, and NF-kappa B signaling pathway. The prominence of these pathways, several of which converge on shared upstream regulators of innate immunity [35,36], suggests that the identified compounds may exert anti-inflammatory effects by modulating central nodes of pattern recognition receptors and cytokine-driven signal transduction. GO biological process analysis (Figure 3B) further supported this, with the terms inflammatory response, response to lipopolysaccharide, and regulation of inflammatory response ranking among the most significantly enriched, together with terms related to reactive oxygen species metabolism and chemokine production, indicating that the overlapping targets are intimately involved in orchestrating innate immune activation and oxidative stress responses that are characteristic of inflammatory pathophysiology [37]. With respect to cellular components (Figure 3C), the targets were predominantly localized to secretory granules and vesicles, the cell surface, membrane rafts, and receptor complexes, consistent with their roles in receptor-mediated signaling and the secretion of inflammatory mediators [38,39,40]. Molecular function analysis (Figure 3D) showed enrichment in nuclear receptor activity, ligand-activated transcription factor activity, and transcription coactivator binding, as well as chemokine receptor activity, MAP kinase activity, and phospholipase-activator activity, reflecting the dual involvement of the overlapping targets in transcriptional regulation and upstream kinase-mediated inflammatory signaling [41]. Therefore, the convergence of KEGG and GO enrichment results across multiple functional dimensions reinforces a multi-target, multi-pathway mechanism whereby the overlapping targets coordinately regulate cytokine and chemokine production, reactive oxygen species generation, and nuclear receptor-mediated transcriptional control, implying the anti-inflammatory potential of primary compounds. On the other hand, network visualization and gene-term relational analyses were conducted to explore the interaction patterns among the overlapping targets and their associated biological functions (Figure 5A,B). The enrichment network (Figure 5A) revealed two major functional clusters: a smaller cluster centered on reactive oxygen species metabolism and cellular response to reactive oxygen species, and a larger, more densely interconnected cluster encompassing inflammatory response, response to lipopolysaccharide, cellular response to biotic stimulus, and chemokine production-related terms [37,42]. Notably, inflammatory response emerged as the most highly connected and largest node within the central position of the network, suggesting that this process serves as a convergent hub through which multiple inflammatory signaling cascades are coordinately regulated. Complementing this, the gene-pathway Sankey diagram and bubble plot (Figure 3B) illustrates the distribution of individual genes across enriched biological process terms, with inflammatory response showing the highest gene ratio and count, alongside the most statistically significant enrichment (−log10 *p* value), consistent with its centrality in the network. TNF was significantly enriched in multiple inflammation-related terms, including inflammatory response, regulation of inflammatory response, and response to lipopolysaccharide, among the targets. This observation highlights TNF’s role as a key hub gene linking innate immune activation to downstream cytokine and chemokine signaling. This high degree of connectivity positions TNF as a potentially critical mediator through which the overlapping targets exert coordinated regulatory effects on inflammatory disease pathogenesis [43]. The convergence of network topology and gene-term association analyses underscores that the anti-inflammatory potential of the overlapping targets is mechanistically anchored in TNF-centered inflammatory signaling. Therefore, molecular docking was subsequently performed to assess the binding affinities and interaction modes between major constituents and the key hub gene (TNF), providing a structural basis for prioritizing TNF as the target for downstream experimental validation. The docking results illustrated that the selected compounds interacted with key residues and had strong binding affinity with the TNF protein.

To experimentally validate this computationally predicted target, we subsequently evaluated whether the *P. pyrifolia* extract could modulate TNF activity and its downstream signaling in a cell-based inflammation model. In vitro assays confirmed that the *P. pyrifolia* extract reduced TNF-α production in LPS-stimulated RAW264.7 cells, thereby verifying the TNF role identified through network pharmacology and its predicted binding interaction from molecular docking. To further clarify the mechanism underlying this suppression of TNF-α, we examined the canonical upstream signaling pathways known to regulate TNF-α transcription. Because prevention of IκBα degradation is critical for blocking NF-κB nuclear translocation, we assessed IκBα stability and found that the *P. pyrifolia* extract inhibited IκBα degradation, thereby reducing nuclear translocation of NF-κB and suppressing downstream transcription of TNF-α. In parallel, the extract decreased phosphorylation of ERK, p38, and JNK, indicating a more generalized suppressive effect on the upstream MAPK signaling cascade that also converges on inflammatory gene transcription. The results suggest that the *P. pyrifolia* extract suppressed the transcription activation of pro-inflammatory genes via the modulation of both NF-κB and MAPK pathways, which is consistent with the TNF-centric signaling network predicted by our computational analysis.

The combination of in silico approaches and experimental validation suggests that the anti-inflammatory effect of *P. pyrifolia* is due to the presence of active compounds that can alleviate inflammatory disorders. The current study highlights *P. pyrifolia* as a promising natural source of bioactive compounds that may be effective in treating inflammation-related diseases.

In addition to the results obtained by the combined experimental and computational investigations, there are some limitations of this work that need to be noted. The anti-inflammatory activity of the *P. pyrifolia* extract and its constituent metabolites were only tested in vitro in cell-based models. Metabolomics-guided network pharmacology analysis, molecular docking, and downstream signaling validation (TNF, NF-κB, and MAPK) provide converging mechanistic evidence; however, these findings need to be confirmed in an appropriate in vivo model (e.g., LPS- or DSS-induced inflammation models) to establish the physiological relevance, systemic bioavailability, and metabolic fate of the active compounds under whole-organism conditions. Meanwhile, this study selected TNF and its downstream NF-κB/MAPK signaling axis as the main hub target. However, other possibly relevant inflammatory or antioxidant-related pathways that are not experimentally verified in the network pharmacology study are worth future exploration. Future studies with in vivo validation and complementary multi-omics approaches (transcriptomics or proteomics) will be needed to fully elucidate the systemic anti-inflammatory mechanisms of the *P. pyrifolia* extract and to support its potential application as a functional product for the improvement of human health.

## 5. Conclusions

This study provides a comprehensive characterization of the anti-inflammatory potential of *P. pyrifolia* by integrating UHPLC-MS/MS-based molecular networking with network pharmacology and molecular docking. Our chemical profiling revealed a diverse landscape of phytochemicals, which were computationally predicted to interact with key inflammatory hubs, most notably the TNF-α signaling pathway. Molecular docking simulations further substantiated these interactions, demonstrating high binding affinities between the identified lead compounds and their corresponding molecular targets. These in silico predictions were robustly validated by in vitro and Western blot experiments, where the *P. pyrifolia* extract significantly attenuated LPS-induced TNF-α production in RAW 264.7 macrophages through IκBα NF-κB, ERK, p38, and JNK MAPK signaling pathways. Collectively, our findings elucidate a multi-component and multi-target mechanism of action, providing a rigorous scientific basis for the traditional use of *P. pyrifolia* in managing inflammatory disorders. While this study establishes a strong mechanistic foundation, further investigations involving in vivo models and pharmacokinetic assessments are warranted to fully translate these pear-derived bioactive compounds into evidence-based therapeutic agents for human health.

## Data Availability

All data is contained within the article.

## Supplementary Materials

The following supporting information can be downloaded at https://www.mdpi.com/article/10.3390/antiox15101274/s1. Table S1: Identification of components from *P. pyrifolia* extract; Table S2: Mass data of 22 primary compounds identified in *P. pyrifolia* peel; Figure S1: Visualization of interactions of ligands and residues; Figure S2: Toll-like receptor signaling pathway; Figure S3: Antioxidative effect of *P. pyrifolia* extract.

## Abbreviations

ABTS	2,2′-Azino-bis(3-ethylbenzothiazoline-6-sulfonic acid
CTL	Control without LPS or sample addition
DG	Degree score
DMEM	Dulbecco’s modified eagle medium
DPPH	2,2-Diphenyl-1-picrylhydrazyl
ERK	Extracellular signal-regulated kinase
GO	Gene Ontology
IκBα	Inhibitor of nuclear factor Kappa B alpha
IL	Interleukin
JNK	c-Jun N-terminal kinase
KEGG	Kyoto encyclopedia of genes and genomes
LC	Liquid chromatography
LPS	Lipopolysaccharide
MAPK	Mitogen-activated protein kinase
MCC	Maximal clique centrality score
MS/MS	Tandem mass spectrometry
NF-κB	Nuclear Factor kappa-light-chain-enhancer of activated B cells
PPI	Protein–protein interaction
*P. pyrifolia*	*Pyrus pyrifolia* (Burm. f.) Nakai
RAW264.7	*Macrophage cell line*
TNF	Tumor necrosis factor
UHPLC	Ultra-high-pressure liquid chromatography
