# Supplementary material for: Integrated UHPLC-MS/MS Metabolomics, Network Pharmacology, Molecular Docking, and Experimental Validation Reveal the Anti-Inflammatory Mechanisms of Pyrus pyrifolia Peel Extract
Source: Antioxidants (Basel). 2026 Oct 2;15(10):1274. doi: 10.3390/antiox15101274 (PMC13700694; doi:10.3390/antiox15101274)
Supplement: Supplementary file 1 [file antioxidants-15-01274-s001.zip › antioxidants-4516626-supplementary.pdf]

Integrated UHPLC-MS/MS Metabolomics, Network Pharmacology, Molecular Docking, and Experimental Validation Reveal the Anti-Inflammatory Mechanisms of *Pyrus pyrifolia* Peel Extract

Ducdat Le [1,2,3,†], Dong Young Rhyu [4,5,†] and Mina Lee [1,2,3,6,*]

1 College of Pharmacy and Research Institute of Life and Pharmaceutical Sciences, Sunchon National University, Suncheon 57922, Jeonnam-Gwangju, Republic of Korea; ddle@scnu.ac.kr
2 Department of Natural Cosmetics Science and AI Digital Bioscience Research Institute, Sunchon National University, Suncheon 57922, Jeonnam-Gwangju, Republic of Korea
3 AI Bio Autonomous Research Core Facility Center, Sunchon National University, Suncheon 57922, Jeonnam-Gwangju, Republic of Korea
4 School of Food and Pharmaceutical Engineering, Mokpo National University, Mokpo 58554, Jeonnam-Gwangju, Republic of Korea; rhyudy@mokpo.ac.kr
5 Department of Biomedicine, Health & Life Convergence Sciences, BK21 FOUR, Mokpo National University, Mokpo 58554, Jeonnam-Gwangju, Republic of Korea
6 Honam Regional Future Drug Development Convergence Education & Research Division (BK21 FOUR), Sunchon National University, Suncheon 57922, Jeonnam-Gwangju, Republic of Korea
* Correspondence: minalee@sunchon.ac.kr or minalee@scnu.ac.kr; Tel.: +82-61-750-3764; Fax: +82-61-750-3708
† These authors contributed equally to this work.

1. Analytical conditions and metabolomic analysis

The *P. pyrifolia* extract was diluted to a final concentration of 10 mg/mL in 50% high grade methanol in water. The solution was filtered using a 0.22 μm PTFE membrane filter (Agilent Technologies, Santa Clara, CA, USA) before LC-MS/MS analysis. Analysis was performed using a Vanquish UHPLC system linked to an Orbitrap Exploris 120 mass spectrometer (Thermo Fisher Scientific, Waltham, MA, USA). Chromatographic separation was performed on a Waters Acquity UPLC HSS T3 column (4.6 × 100 mm, 1.8 μm, Waters, Milford, MA, USA) at 40 °C. The flow rate was 0.2 mL/min and the injection volume was 4 μL. The gradient mobile phase was solvent A (0.1% formic acid in water) and solvent B (0.1% formic acid in acetonitrile) buffering with 0.01% ammonia applied as follows: 8-15% B (0-4 min), 15-32% B (4-8 min), 32-53% B (8-11 min), 53-100% B (11-24 min), and held for 3 min, returning to 8% B over 1 min for re-equilibration. The MS conditions were as follows: spay voltage positive and negative (3500 V and 2500 V); sheath gas (N_2) 50; aux gas (N_2) 15; ion transfer tube temperature 320 °C; vaporizer temperature 350 °C; HCD collision energies (%) 15, 30, and 60; Orbitrap resolution 120,000; scan range 100-1500 m/z. Calibration of the Orbitrap was performed by infusion of the calibration solution with the spray parameters adjusted to obtain a spray stability of < 15 % RSD. Raw Orbitrap MS/MS data files were then processed using MZmine version 3.9.0 prior to uploading to the GNPS platform (University of California, San Diego, La Jolla, CA, USA). The software MS-DIAL (Riken Center for Sustainable Resource Science, Yokohama City, Kanagawa, Japan) was used to perform further analysis with association with multiple mass databases (American mass databases, HMDB, etc). Characteristic precursor ions and their fragmentation patterns were used for the identification of peaks in chromatograms. The threshold was defined as: MS1: 0.0005 Da; MS2: 0.0001 score cut-off: 700 (0.7). The FBMN-GNPS2 was used for compound identification by matching features with several mass spectrum databases online based on isotope patterns and mass duplication. A similarity matrix was created and the cosine score factor was used to keep structurally relevant connections and visualization.

2, DPPH and ABTS assays

The antioxidant activity of *P. pyrifolia* extract was determined by the scavenging ability of sample on DPPH radical (Sigma-Aldrich, St Louis, MO, USA). Each well contained 100 μL of the sample volume diluted in ethanol at final concentrations (10 and 100 μg/mL) and 100 μL of DPPH (200 μM) in ethanol solution. Control was prepared under the same conditions with the amount of sample replaced by addition of ethanol. All wells were mixed well and incubated at room temperature for 30 min in shade. When DPPH reacted with an antioxidant sample, the color change from deep violet to bright yellow was measured at 517 nm using a micro reader (Epoch, Biotek Instruments, Winooski, VT, USA). Additionally, 2.45 mM ABTS (Sigma-Aldrich, Co.) was

mixed with 7 mM 2,2′-azobis(2-aminopropane) dihydrochloride and incubated for 16 h at 4 °C. The sample (100 μL) and ABTS solution (100 μL) were combined and reacted at room temperature for 20 min before being put on 96-well plates. Absorbance was recorded at 734 nm. Ascorbic acid (Sigma-Aldrich, Co., St. Louis, MO, USA) (10 and 100 μg/mL) was used as positive control in both experiments. Results were expressed as a percentage of DPPH and ABTS reduction of treated samples against control wells. The antioxidant activities of *P. pyrifolia* extract against radicals were obtained as follows:

% Radical scavenging activity = (A control − A sample) × 100/ (A control)

A sample = absorbance of the sample, A control = absorbance of untreated sample.

Table S1. Identification of components from *P. pyrifolia* extract.

No.	Compound	RT (min)	Formula	Adduct	*m/z* (Da)	Error (mDa)	Class
1	Unknown	1.088	-	$[M+H]^+$	128.0192	-	-
2	Unknown	1.088	-	$[M+H]^+$	241.9994	-	-
3	2-Hydroxy-3,4-dimethoxybenzoic acid	1.246	$C_9H_{10}O_5$	$[M+Na]^+$	221.0418	0.183	Organic acids
4	Gentiobiose	1.285	$C_{12}H_{22}O_{11}$	[M+K]+	381.0787	0.641	Terpenoids
5	D-Fructose	1.334	$C_6H_{12}O_6$	$[M+NH_4]^+$	180.0864	0.0004	Glycosides
6	D-1-[(3-Carboxypropyl)amino]-1-deoxyfructose	1.372	$C_{10}H_{19}NO_7$	$[M+H]^+$	266.1230	0.003	Alkaloids
7	D-Sorbitol	1.420	$C_6H_{14}O_6$	$[M+H]^+$	183.0861	0.0001	Glycosides
8	Mycosporine serinol	1.699	$C_{11}H_{19}NO_6$	$[M+H]^+$	262.1280	0.031	Phenylpropanoids
9	Unknown	1.699	-	$[M+H]^+$	280.1386	-	-
10	Tyrosine	1.894	$C_9H_{11}NO_3$	$[M+H]^+$	182.0808	0.351	Alkaloids
11	Noradrenaline	1.952	$C_8H_{11}NO_3$	$[M+H-H_2O]^+$	152.0702	0.366	Alkaloids
12	Methyl 3,4,5-trihydroxycyclohexene-1-carboxylate	1.962	$C_8H_{12}O_5$	$[M +H\_ACN]^+$	230.1020	0.29	Phenylpropanoids
13	Norleucine	2.178	$C_6H_{13}NO_2$	$[M+H]^+$	132.1018	0.076	Alkaloids
14	Methoxyindoleacetic acid	2.250	$C_11H_{11}NO_3$	$[M+H]^+$	206.0810	0.9	Alkaloids
15	L-Prolyl-L-isoleucine	2.379	$C_{11}H_{20}N_2O_3$	$[M+H]^+$	229.1544	1.064	Alkaloids
16	Hydroquinone	2.492	$C_6H_6O_2$	$[M+H]^+$	111.04394	0.114	Phenols
17	L-Tyrosine	2.522	$C_9H_{11}NO_3$	$[M+H]^+$	180.0653	0.244	Alkaloids
18	Unknown	2.571	-	$[M+H]^+$	308.1338	-	-
19	2-Benzyl-1-methyl-1,4-dihydroquinazolin-4-one	2.582	$C_{16}H_{14}N_2O$	$[M+H]^+$	273.0963	0.5353	Alkaloids
20	Unknown	2.925	-	$[M+H]^+$	398.1287	-	-
21	5,7-Dihydroxycoumarin	3.016	$C_9H_6O_4$	$[M+H]^+$	179.0337	0.198	Phenylpropanoids
22	2-Oxindole	3.201	C_8H_7NO	$[M+H]^+$	134.0599	0.107	Alkaloids
23	Indole-2-carboxylic acid	3.201	$C_9H_7NO_2$	$[M+NH_4]^+$	179.0812	0.275	Alkaloids
24	5-hydroxy-tryptophan	3.250	$C_{11}H_{12}N_2O_3$	$[M+H]^+$	219.0737	0.893	Alkaloids
25	1-Methyladenosine	3.624	$C_{11}H_{15}N_5O_4$	$[M+H]^+$	282.1192	0.488	Alkaloids

26	[(2*S*,3*R*,4*S*,5*S*,6*R*)-3,4,5-trihydroxy-6-(hydroxymethyl)oxan-2-yl] (*E*)-3-phenylprop-2-enoate	3.740	$C_{15}H_{18}O_7$	$[M+NH_4]^+$	328.1388	0.214	Glycosides
27	Methyl 2-(4*a*,6,10*a*-trihydroxy-1-methyl-5,10-dioxo-3,4-dihydro-1H-benzo[g]isochromen-3-yl)acetate	3.891	$C_{17}H_{18}O_8$	$[M+NH_4]^+$	368.1336	0.427	Naphthopyranones
28	Phenylalanine	4.038	$C_9H_{11}NO_2$	$[M+H]^+$	166.0861	0.137	Alkaloids
29	Indoline	4.050	C_8H_9N	$[M+H]^+$	120.0807	0.061	Alkaloids
30	Pyrogallol	4.431	$C_6H_6O_3$	$[M+H]^+$	127.0389	0.114	Phenols
31	N-Acetylleucine	4.453	$C_8H_{15}NO_3$	$[M+H]^+$	196.0966	0.2469	Fatty Acids
32	Pantothenic acid	4.495	$C_9H_{17}NO_5$	$[M+H]^+$	220.1177	0.32	Alkaloids
33	3-[2-Methoxy-3-[(2*S*, 3*R*, 4*S*, 5*S*, 6*R*)-3,4,5-trihydroxy-6-(hydroxymethyl)oxan-2-yl]oxyphenyl]propanoic acid	4.962	$C_{16}H_{22}O_9$	$[M+NH_4]^+$	376.1598	0.458	Glycosides
34	Piperic acid	5.395	$C_{12}H_{10}O_4$	$[M+NH_4]^+$	236.0916	0.168	Fatty Acids
35	Methyl Asterrate	5.586	$C_{18}H_{18}O_8$	$[M+NH_4]^+$	380.1337	0.336	Phenols
36	4,7,8-Trimethoxyfuro[2,3-b]quinoline	5.999	$C_{14}H_{13}NO_4$	$[M+H]^+$	260.0915	0.244	Alkaloids
37	5-Phenyllevulinic acid	6.062	$C_{11}H_{12}O_3$	$[M+NH_4]^+$	210.1122	0.244	Organic acids
38	(*Z*)-3-[4-methoxy-2-[(2*S*,3*R*,4*S*,5*S*,6*R*)-3,4,5-trihydroxy-6-(hydroxymethyl)oxan-2-yl]oxyphenyl]prop-2-enoic acid	6.253	$C_{16}H_{20}O_9$	$[M+NH_4]^+$	374.1441	0.0009	Glycosides
39	4-(2-Methyl-6-oxopyran-3-yl)butanoic acid	6.612	$C_{10}H_{12}O_4$	$[M+H]^+$	197.0807	0.168	Pyranone
40	(*E*)-9-(1,3-benzodioxol-5-yl)-1-piperidin-1-ylnon-8-en-1-one	6.629	$C_{21}H_{29}NO_3$	$[M+H]^+$	344.2273	1.272	Alkaloids
41	7-Hydroxy-coumarin	6.746	$C_9H_6O_3$	$[M+H]^+$	163.0388	0.168	Phenylpropanoids
42	Chlorogenic acid	6.789	$C_{16}H_{18}O_9$	$[M+H]^+$	355.1021	0.0011	Polyphenols
43	(*E*)-3-[4-[(2*S*,3*R*,4*S*,5*S*,6*R*)-3,4,5-trihydroxy-6-(hydroxymethyl)oxan-2-yl]oxyphenyl]prop-2-enoic acid	6.844	$C_{15}H_{18}O_8$	$[M+NH_4]^+$	344.1337	0.0003	Glycosides
44	5-Aminolevulinic acid	7.014	$C_5H_9NO_3$	$[M+Na]^+$	154.0497	0.061	Alkaloids
45	4-Methoxy-6-prop-2-enyl-1,3-benzodioxole	7.101	$C_{11}H_{12}O_3$	$[M+H]^+$	193.0857	0.183	Phenylpropanoids
46	Unknown	7.154	-	$[M+H]^+$	388.2535	-	-
47	2-[(2-Amino-3-methylbutanoyl)amino]-3-phenylpropanoic acid	7.154	$C_{14}H_{20}N_2O_3$	$[M+H]^+$	265.1543	0.0003	Alkaloids
48	Cryptotanshinone	7.185	$C_{19}H_{20}O_3$	$[M+H]^+$	297.1443	0.2111	Terpenoids
49	(*E*)-3-(3,4,5-trimethoxyphenyl)prop-2-enal	7.196	$C_{12}H_{14}O_4$	$[M+H]^+$	223.0962	0.259	Aldehydes
50	Roseoside	7.544	$C_{19}H_{30}O_8$	$[M+H]^+$	387.2008	0.0022	Glycosides
51	Sinapoyl malate	7.545	$C_{15}H_{16}O_9$	$[M+H-C_4H_6O_5]^+$	207.1375	0.427	Organic acids
52	4-[4-(*β*-D-glucopyranosyloxy)-2-hydroxy-2,6,6-trimethylcyclohexylidene]-3-Buten-2-one	7.545	$C_{19}H_{30}O_8$	$[M+Na]^+$	409.1824	0.854	Terpenoids
53	(*E*)-3-(4-hydroxy-3-methoxyphenyl)prop-2-enamide	7.826	$C_{10}H_{11}NO_3$	$[M+H]^+$	194.0810	0.198	Alkaloids
54	8-Methoxypsoralen	7.826	$C_{12}H_8O_4$	$[M+NH_4]^+$	234.0735	1.079	Furocoumarins
55	Abieta-8(14),9(11),12-triene-7,18-diol	7.847	$C_{20}H_{30}O_2$	$[M+H+ACN]^+$	344.2540	0.3852	Diterpenoids

56	Pyroglutamylisoleucine	8.015	$C_{11}H_{18}N_2O_4$	$[M+H]^+$	243.1337	0.32	Dipeptides
57	2,3,8,9,10-Pentamethoxy-6a,11a-dihydro-6H-[1]benzofuro[3,2-c]chromene	8.036	$C_{20}H_{22}O_7$	$[M+H]^+$	375.1433	0.488	Benzofurochromene
58	(7*E*)-11-Hydroxy-3-isobutyl-4,5,8,13,13-pentamethyl-3,3a,4,6a,9,10,11,11a,14a,15-decahydro-1H-[1,3]dioxolo[9,10]oxacyclododecino[2,3-d]isoindole-1,16(2H)-dione	8.095	$C_{27}H_{41}NO_6$	$[M+H]^+$	476.3053	2.069	Macrolide
59	4'-Hydroxy-4-methoxydalbergione	8.185	$C_{16}H_{14}O_4$	$[M+H]^+$	271.0962	0.214	Flavonoids
60	Miriquidic acid	8.185	$C_{25}H_{30}O_8$	$[M+Na]^+$	481.1851	1.099	Organic acids
61	Selaginpulvilin T	8.185	$C_{36}H_{28}O_5$	$[M+H]^+$	539.1882	1.16	Polyalcohols
62	3-Phenyllactic acid	8.369	$C_9H_{10}O_3$	$[M+H]^+$	167.0702	0.107	Organic acids
63	2-[(1*R*,2*R*)-3-oxo-2-[(*Z*)-5-[3,4,5-trihydroxy-6-(hydroxymethyl)oxan-2-yl]oxypent-2-enyl]cyclopentyl]acetic acid	8.567	$C_{18}H_{28}O_9$	$[M+H]^+$	389.1780	1.755	Fatty acyl glycosides
64	Norcocaine	8.580	$C_{16}H_{19}NO_4$	$[M+H]^+$	290.1384	0.366	Alkaloids
65	3,5-Dihydroxydecanoic acid	8.591	$C_{10}H_{20}O_4$	$[M+H]^+$	205.1433	0.107	Fatty acids
66	Indole-3-pyruvic acid	8.649	$C_{11}H_9NO_3$	$[M+H]^+$	204.0654	0.137	Alkaloids
67	Argaminolic A	8.649	$C_{11}H_{11}NO_4$	$[M+H]^+$	222.0759	0.076	Amino phenolics
68	Sweroside	8.717	$C_{16}H_{22}O_9$	$[M+Na]^+$	381.1175	1.423	O-glycosyl compounds
69	4-(Hydroxymethyl)-1-isopropyl-3-cyclohexen-1-yl beta-D-glucopyranoside	8.728	$C_{16}H_{28}O_7$	$[M+Na]^+$	355.1724	0.361	Terpenoids
70	4-(Beta-D-glucopyranosyloxy)-6-methoxy-5-Benzofuranpropanoic acid	8.728	$C_{18}H_{22}O_{10}$	$[M+Na]^+$	421.1099	0.58	Benzofurans
71	(6*E*)-14-hydroxy-4,14,16-trimethyl-8-methylidene-17-tridecyl-1-oxa-4,9,12-triazacycloheptadec-6-ene-2,5,10,13,15-pentone	8.816	$C_{30}H_{49}N_3O_7$	$[M+H]^+$	564.3583	1.648	Alkaloids
72	Cinnamic acid	8.843	$C_9H_8O_2$	$[M+H]^+$	149.0596	0.412	Cinnamates
73	2-Phenylethyl-galactoside	8.878	$C_{14}H_{20}O_6$	$[M+NH_4]^+$	302.1596	0.0004	Glycosides
74	Unknown	8.893	-	$[M+H]^+$	338.1806	-	-
75	Coniferin	8.893	$C_{16}H_{22}O_8$	$[M+H]^+$	343.1362	1.533	Cinnamates
76	8-Hydroxypinoresinol-4'-*O*-β-D-Glucopyranoside	9.114	$C_{26}H_{32}O_{12}$	$[M+NH_4]^+$	554.2226	0.0004	Glycosides
77	Hydroxyphenyllactate	9.310	$C_{11}H_8O_4$	$[M+Na]^+$	205.0493	0.2493	Ketones
78	8-Hydroxy-3-methyl-1-oxo-3,4-dihydroisochromene-5-carboxylic acid	9.310	$C_{11}H_{10}O_5$	$[M+H]^+$	223.0598	0.275	Phenylpropanoids
79	4-(Hydroxymethyl)-1-isopropyl-3-cyclohexen-1-yl β-D-glucopyranoside	9.310	$C_{16}H_{28}O_7$	$[M+Na]^+$	355.1723	0.397	Glycosides
80	2-(3,4-Dihydroxyphenyl)-5-hydroxy-7-[(2*S*,3*R*,4*S*,5*S*,6*R*)-3,4,5-trihydroxy-6-[[(2*R*,3*R*,4*R*,5*R*,6*S*)-3,4,5-trihydroxy-6-methyloxan-2-yl]oxymethyl]oxan-2-yl]oxychromen-4-one	9.448	$C_{27}H_{30}O_{15}$	$[M+H]^+$	595.1654	0.0004	Flavonoids
81	Villol	9.668	$C_{23}H_{22}O_9$	$[M+NH_4]^+$	460.1596	0.549	Flavonoids
82	{(1*R*,2*R*)-2-[(2*Z*)-5-Hydroxy-2-penten-1-yl]-3-oxocyclopentyl}acetic acid	9.704	$C_{13}H_{14}N_4$	$[M+Na]^+$	249.1118	1.09	Alkaloids

83	Unknown	9.916	-	$[M+H]^+$	552.2068	-	-
84	(6*S*,7*aR*)-5,6,7,7*a*-tetrahydro-6-hydroxy-4,4,7*a*-trimethyl-2(4H)-Benzofuranone	10.176	$C_{11}H_{16}O_3$	$[M+Na]^+$	219.1014	0.843	Benzofurans
85	Perillyl alcohol	10.432	$C_{10}H_{16}O$	$[M+H]^+$	153.1272	0.229	Terpenoids
86	Neodiosmin	10.606	$C_{28}H_{32}O_{15}$	$[M+H]^+$	609.1808	0.0032	Flavonoids
87	8-(2-Hydroxypropan-2-yl)furo[2,3-h]chromen-2-one	10.793	$C_{14}H_{12}O_4$	$[M+H]^+$	245.0806	0.259	Coumarins
88	5,7-Dihydroxy-2-(4-hydroxy-3-methoxyphenyl)-3-[3,4,5-trihydroxy-6-(hydroxymethyl)oxan-2-yl]oxychromen-4-one	11.017	$C_{22}H_{22}O_{12}$	$[M+H]^+$	479.1177	0.0003	Glycosides
89	4,5-Dihydro-2',3'-epoxy-15-deoxygoyazensolide	11.401	$C_{19}H_{22}O_8$	$[M+Na]^+$	401.1204	0.305	Terpenoids
90	[4,8-Dihydroxy-3-(3-methoxy-3-oxoprop-1-en-2-yl)-8a-methyl-5-oxo-1,2,3,4,4a,6,7,8-octahydronaphthalen-2-yl] 3,4-dihydroxy-2-methylidenebutanoate	11.808	$C_{20}H_{28}O_9$	$[M+Na]^+$	435.1644	1.587	Organic acids
91	3-Indoleacrylic acid	12.141	$C_{11}H_9NO_2$	$[M+H]^+$	188.0705	0.092	Organic acids
92	2-Methoxy-1,4-naphthoquinone	12.270	$C_{11}H_8O_3$	$[M+H]^+$	189.0544	0.183	Naphthoquinones
93	Abscisic acid	12.594	$C_{15}H_{20}O_4$	$[M+H-H_2O]^+$	247.1325	0.0005	Organic acids
94	Unknown	12.678	$C_{14}H_{22}N_6O_6$	$[M+H]^+$	371.1673	0.092	Alkaloids
95	(3a*R*,6*R*,7a*R*)-6-methyl-3-methylidene-6-(4-oxopentyl)-3a,4,7,7a-tetrahydro-1-benzofuran-2,5-dione	13.484	$C_{15}H_{20}O_4$	$[2M+Na]^+$	551.2609	2.136	Terpenoids
96	9_10-*α*-Epoxy-eremanthin	13.496	$C_{15}H_{18}O_3$	$[M+H]^+$	247.1326	0.275	Gamma butyrolactones
97	(4*R*)- dihydro-3-methylene-4-[(2E)-3-methyl-4-(tetrahydro-4-methyl-5-oxo-2-furanyl)-2-buten-1-yl]-2(3H)-Furanone	13.496	$C_{15}H_{20}O_4$	$[M+Na]^+$	287.1250	0.336	Terpenoids
98	3-(Hexopyranosyloxy)-2-[(2*Z*)-2-penten-1-yl]-cyclopentaneacetic acid	13.699	$C_{18}H_{30}O_8$	$[M+Na]^+$	397.1829	0.366	Glycosides
99	3,4-Dihydro-6-hydroxy-8-methoxy-3-methyl-1H-2-Benzopyran-1-one	14.325	$C_{11}H_{12}O_4$	$[M+H]^+$	209.0806	0.229	Benzopyrans
100	Neobavaisoflavone	14.325	$C_{20}H_{18}O_4$	$[2M+Na]^+$	667.2358	1.859	Flavonoids
101	Syringaresinol	14.612	$C_{22}H_{26}O_8$	$[M+H]^+$	417.1538	1.19	Polyphenols
102	Unknown	14.612	-	$[M+H]^+$	637.2249	-	-
103	Unknown	14.851	-	$[M+H]^+$	429.2817	-	-
104	Sparteine-15-carboxylic acid	14.875	$C_{16}H_{26}N_2O_2$	$[M+H]^+$	279.2040	1.06	Quinolizines
105	Unknown	14.875	-	$[M+H]^+$	257.2221	-	-
106	9-Oxootre	15.465	$C_{18}H_{28}O_3$	$[M+H-H_2O]^+$	275.2003	0.0003	Fatty acids
107	(10*E*,15*E*)-9,12,13-trihydroxyoctadeca-10,15-dienoic acid	15.478	$C_{18}H_{32}O_5$	$[M+H]^+$	351.2140	0.031	Fatty acids
108	9-Methoxycarbonyldec-9-enoic acid	15.568	$C_{12}H_{20}O_4$	$[M+H]^+$	229.1432	0.275	Fatty acids
109	Unknown	15.574	-	$[M+H]^+$	211.0751	-	-
110	Dipropyl phthalate	15.585	$C_{14}H_{18}O_4$	$[M+H]^+$	251.1251	0.273	Organic acids
111	Unknown	15.609	-	$[M+H]^+$	607.2146	-	-
112	9(10)-EpOME	16.165	$C_{18}H_{32}O_3$	$[M+H-H_2O]^+$	279.2316	0.0004	Fatty acids

113	8-Hydroxy-8-(3-octyloxiran-2-yl)octanoic acid	16.165	$C_{18}H_{3}4O_{4}$	$[M+H-H_2O]^+$	297.2422	0.183	Fatty acids
114	5-[5-Hydroxy-3-(hydroxymethyl)pentyl]-8a-(hydroxymethyl)-5,6-dimethyl-3,4,4a,6,7,8-hexahydronaphthalene-1-carboxylic acid	16.165	$C_{20}H_{34}O_5$	$[M+H]^+$	355.2453	1.704	Terpenoids
115	(*Z*)-9,12,13-trihydroxyoctadec-15-enoic acid	16.514	$C_{18}H_{34}O_5$	$[M+NH_4]^+$	348.2741	1.343	Fatty acids
116	FA 18:3+1O	16.525	$C_{18}H_{30}O_3$	$[M+H]^+$	295.2264	1.045	Fatty acids
117	(9*Z*)-5,8,11-Trihydroxyoctadec-9-enoic acid	16.525	$C_{18}H_{34}O_5$	$[M+Na]^+$	353.2294	1.391	Fatty acids
118	Lauryldiethanolamine	19.443	$C_{16}H_{35}NO_2$	$[M+H]^+$	274.2738	0.519	Alkaloids
119	Gelomulide N	21.540	$C_{24}H_{32}O_7$	$[M+H-H_2O]^+$	415.2109	0.61	Naphthofurans
120	7b,9-Dihydroxy-3-(hydroxymethyl)-1,1,6,8-tetramethyl-5-oxo-1,1a,1b,4,4a,5,7a,7b,8,9-decahydro-9aH-cyclopropa[3,4]benzo[1,2-e]azulen-9a-yl acetate	21.540	$C_{22}H_{30}O_6$	$[M+H+ACN]^+$	432.2375	0.427	Terpenoids

Table S2. Mass data of 22 primary compounds identified from the *P. pyrifolia* peel.

No.	Compound	RT (min)	Formula	Adduct	*m/z* (Da)	MS^n
3	2-Hydroxy-3,4-dimethoxybenzoic acid	1.246	$C_9H_{10}O_5$	$[M+Na]^+$	221.0418	221.0420/205.0557
4	Gentiobiose	1.285	$C_{12}H_{22}O_{11}$	$[M+K]^+$	381.0787	381.0787/342.1391
6	D-1-[(3-carboxypropyl)amino]-1-deoxyfructose	1.372	$C_{10}H_{19}NO_7$	$[M+H]^+$	266.1230	266.1230/248.1224/ 230.1020/146.0652
7	D-Sorbitol	1.420	$C_6H_{14}O_6$	$[M+H]^+$	183.0861	183.0861/147.0649/ 133.0606
8	Mycosporine serinol	1.699	$C_{11}H_{19}NO_6$	$[M+H]^+$	262.1280	262.1280/244.1174
10	Tyrosine	1.894	$C_9H_{11}NO_3$	$[M+H]^+$	182.0808	182.0809/136.0755/ 119.0853
12	Methyl 3,4,5-trihydroxycyclohexene-1-carboxylate	1.962	$C_8H_{12}O_5$	$[M+ACN+H]^+$	230.10201	230.1022/212.0916
11	Noradrenaline	1.952	$C_8H_{11}NO_3$	$[M+H-H_2O]^+$	152.0702	152.0704/136.0756
13	Norleucine	2.178	$C_6H_{13}NO_2$	$[M+H]^+$	132.1018	132.1017/86.0964
14	Methoxyindoleacetic acid	2.250	$C_{11}H_{11}NO_3$	$[M+H]^+$	206.0810	206.0809/146.0600
26	[(2*S*,3*R*,4*S*,5*S*,6*R*)-3,4,5-trihydroxy-6-(hydroxymethyl)oxan-2-yl] (*E*)-3-phenylprop-2-enoate	3.740	$C_{15}H_{18}O_7$	$[M+NH_4]^+$	328.13885	328.1388/165.0757
30	Pyrogallol	4.431	$C_6H_6O_3$	$[M+H]^+$	127.03886	127.0389/109.0284/ 81.0335
31	N-acetylleucine	4.453	$C_8H_{15}NO_3$	$[M+Na]^+$	196.09657	196.0966/183.0861/ 180.0896/147.0650
32	Pantothenic acid	4.495	$C_9H_{17}NO_5$	$[M+H]^+$	220.11763	220.1178/202.1071
35	Methyl asterrate	5.586	$C_{18}H_{18}O_8$	$[M+NH_4]^+$	380.13367	380.1336/262.1071
36	4,7,8-Trimethoxyfuro[2,3-b]quinoline	5.999	$C_{14}H_{13}NO_4$	$[M+H]^+$	260.09149	260.0915/245.0587/ 227.0911/201.0755
37	5-Phenyllevulinic acid	6.062	$C_{11}H_{12}O_3$	$[M+ NH_4]^+$	210.11224	210.1122/
77	Hydroxyphenyllactate	9.31	$C_{11}H_8O_4$	$[M+Na]^+$	205.04929	205.0493/145.0494
117	(9*Z*)-5,8,11-Trihydroxyoctadec-9-enoic acid	16.53	$C_{18}H_{34}O_5$	$[M+H]^+$	353.22943	353.2294/296.2301
85	Perillyl alcohol	10.432	$C_{10}H_{16}O$	$[M+H]^+$	153.12717	153.1272/135.1168
118	Lauryldiethanolamine	19.443	$C_{16}H_{35}NO_2$	$[M+H]^+$	274.27359	274.2740/256.2635
119	Gelomulide N	21.54	$C_{24}H_{32}O_7$	$[M-H_2O+H]^+$	415.21091	415.2010/135.0805/ 119.0856

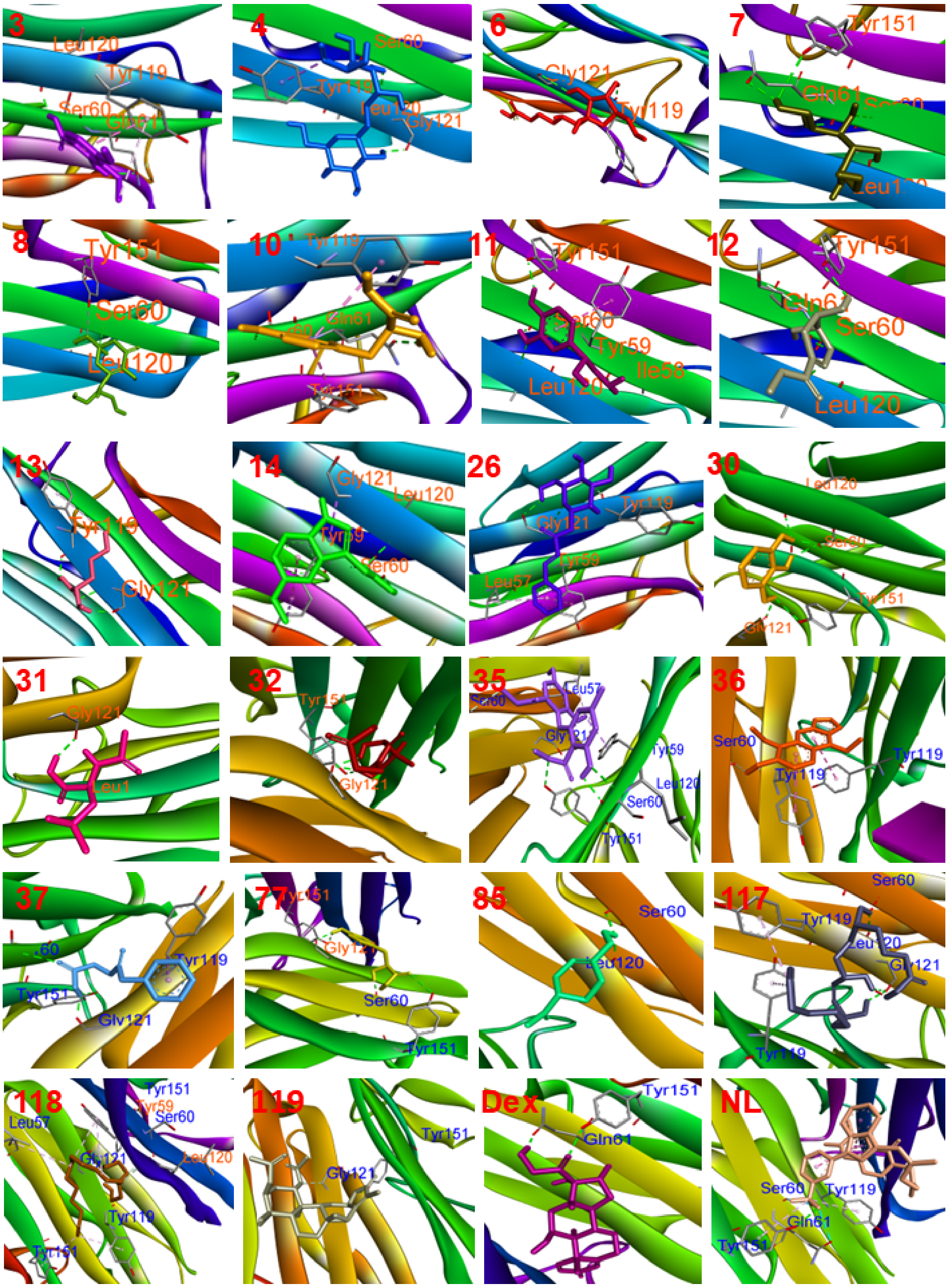

Figure S1. Visualization of interactions of ligands and residues.

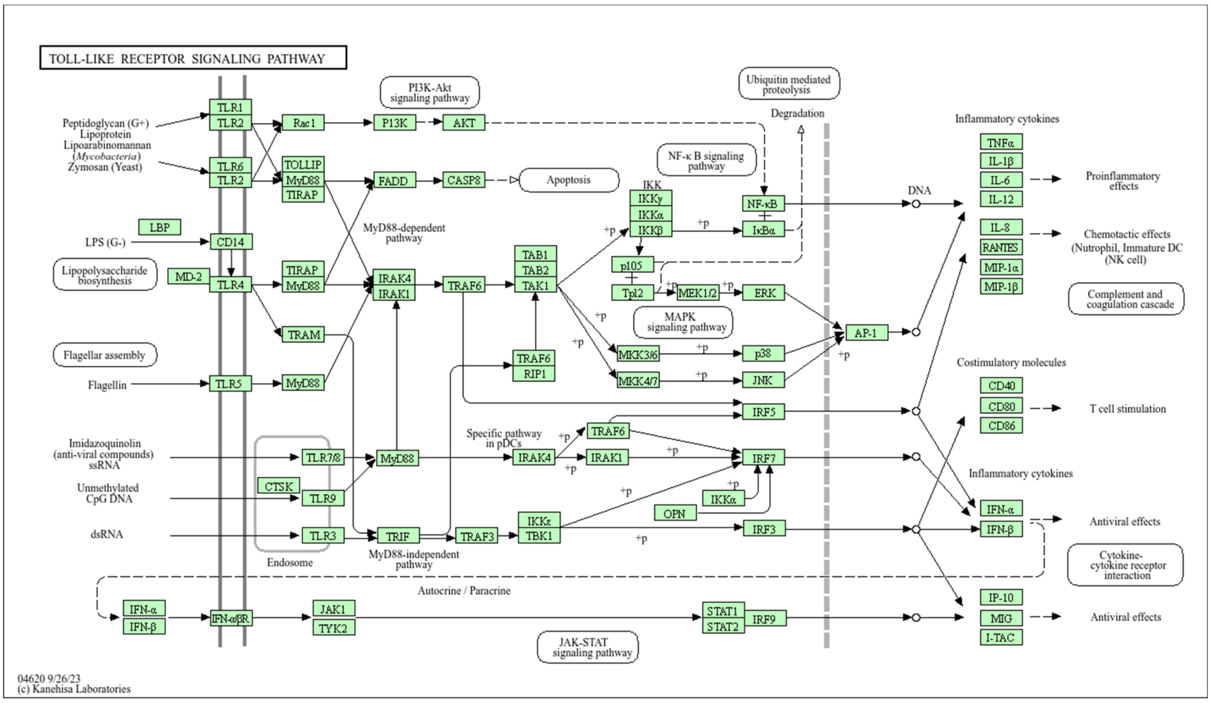

Figure S2. Toll-like receptor signaling pathway.

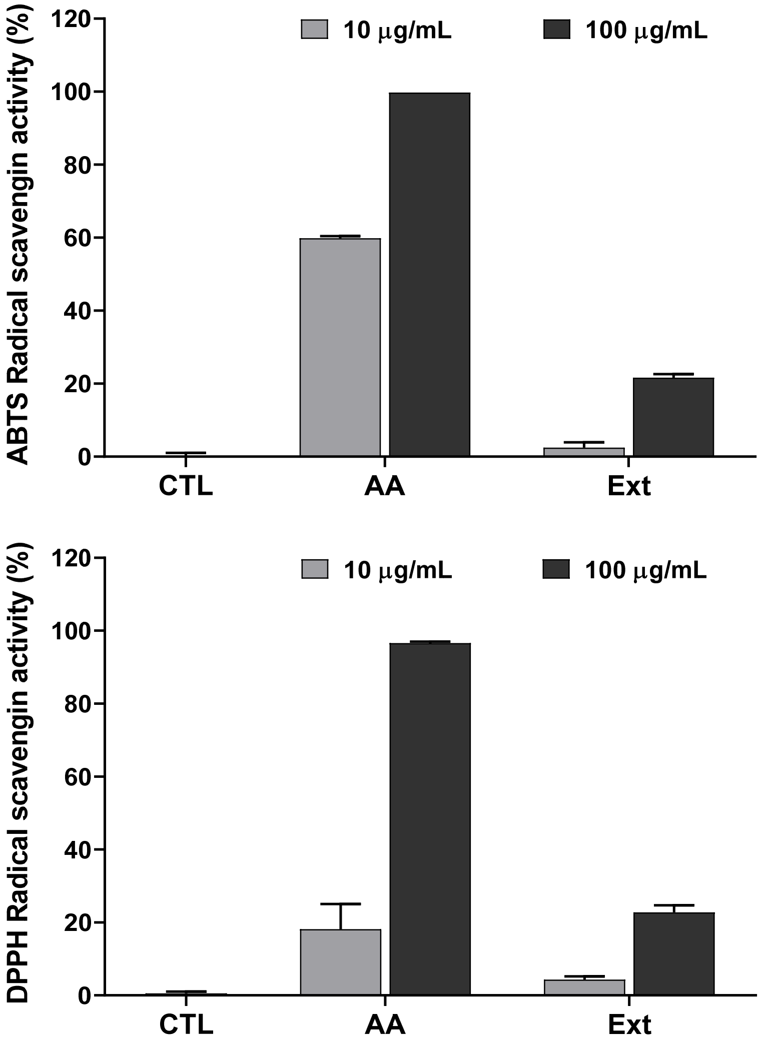

Figure S3. Antioxidative effect of *P. pyrifolia* extract.
